# Pharmacological Basis of *Rumex hastatus* D. Don in Gastrointestinal Diseases with Focusing Effects on H^+^/K^+^-ATPase, Calcium Channels Inhibition and PDE Mediated Signaling: Toxicological Evaluation on Vital Organs

**DOI:** 10.3390/molecules27185919

**Published:** 2022-09-12

**Authors:** Neelum Gul Qazi, Arif-ullah Khan, Sumra Wajid Abbasi, Fawad Ali Shah, Faisal Rasheed, Fawad Ali, Syed Shams ul Hassan, Simona Bungau

**Affiliations:** 1 Riphah Institute of Pharmaceutical Sciences, Riphah International University, Islamabad 46000, Pakistan; 2 Nums Department of Biological Sciences, National University of Medical Sciences, Rawalpindi, Punjab 46000, Pakistan; 3 BreathMAT Lab., Nuclear Medicine, Oncology and Radiotherapy Institute, Islamabad 45320, Pakistan; 4 Department of Pharmacy, Kohat University of Science and Technology, Kohat 26000, Pakistan; 5 Shanghai Key Laboratory for Molecular Engineering of Chiral Drugs, School of Pharmacy, Shanghai Jiao Tong University, Shanghai 200240, China; 6 Department of Natural Product Chemistry, School of Pharmacy, Shanghai Jiao Tong University, Shanghai 200240, China; 7 Department of Pharmacy, Faculty of Medicine and Pharmacy, University of Oradea, 410028 Oradea, Romania

**Keywords:** *Rumex hastatus*, antioxidant, anti-inflammatory, anti-ulcer, H^+^/K^+^-ATPase, calcium channels

## Abstract

This present study aimed to delineate *Rumex hastatus* D. Don crude extract (Rh.Cr), n-Hexane, ethyl acetate, aqueous fractions (Rh.n-Hex, Rh.ETAC, Rh.Aq) and rutin for antidiarrheal, antisecretory effects, anti-spasmodic, gastrointestinal transient time, anti *H. pylori*, antiulcer effects, and toxicology. The preliminary phytochemical analysis of *Rumex hastatus* showed different phytoconstituents and shows different peaks in GC-MC chromatogram. *Rumex hastatus* crude extract (Rh.Cr), fractions, and rutin attributed dose-dependent (50–300 mg/kg) protection (0–100%) against castor oil-induced diarrhea and dose-dependently inhibited intestinal fluid secretions in mice. They decreased the distance traversed by charcoal in the gastrointestinal transit model in rats. In rabbit jejunum preparations, Rh.Cr and Rh.ETAC caused a concentration-dependent relaxation of both spontaneous and K^+^ (80 mM)-induced contractions at a similar concentration range, whereas Rh.n-Hex, rutin, and verapamil were relatively potent against K^+^-induced contractions and shifted the Ca^2+^ concentration–response curves (CRCs) to the right, Rh.Cr (0.3–1 mg/mL) and Rh.ETAC (0.1–0.3 mg/mL) shifted the isoprenaline-induced inhibitory CRCs to the left. Rh.n-Hex, Rh.ETAC and rutin showed anti-*H. pylori* effect, also shows an inhibitory effect against H^+^/K^+^-ATPase. *Rumex hastatus* showed gastroprotective and antioxidant effects. Histopathological evaluation showed improvement in cellular architecture and a decrease in the expression of inflammatory markers such as, cyclooxygenase (COX-2), tumor necrosis factor (TN,F-α) and phosphorylated nuclear factor kappa B (p-NFƙB), validated through immunohistochemistry and ELISA techniques. In RT-PCR it decreases H^+^/K^+^-ATPase mRNA levels. *Rumex hastatus* was found to be safe to consume up to a dose of 2000 mg/kg in a comprehensive toxicity profile. Docking studies revealed that rutin against H^+^/K^+^-ATPase pump and voltage-gated L-type calcium channel showed E-values of −8.7 and −9.4 Kcal/mol, respectively. MD simulations Molecular Mechanics Poisson Boltzmann surface area and molecular mechanics Generalized Born surface area (MMPBSA/GBSA) findings are consistent with the in-vitro, in-vivo and docking results.

## 1. Introduction

Medicinal plants have played a vital role in the development of therapeutic agents, the oldest civilizations having solid contributions in this regard [1,2,3]. These are gifts from nature for countering unlimited series of lethal disorders; thus, medicinal plants are receiving additional deliberation at present than before, particularly in the field of medicine and pharmacology. About 80% of people living in developing countries are still dependent on traditional medicine for their primary health care [4,5]. The bioactive phytochemical components of the plant are under investigation globally for their broad-spectrum medical properties which are strictly linked to their specific chemical structure [6,7]. Medicinal plants are discovered as a foundation to separate unadulterated active compounds or in the form of complex, where there is a synergistic mixture of active constituents and other elements such as enzymes, resins, essential oils, and tannins to expedite their activities. The health endorsing potentials of medicinal plants are typically obtained from the interaction of the constituents present in the plants. Nevertheless, the prominence of the usage of total herbs as medications and food additives is progressively substituting the procedures to segregate the naturally active novel as the leading drug [8,9].

There are 250 species of herbs that are present in the genus *Rumex*. *Rumex hastatus* D. Don, commonly known as toothed dock, is the prime species that has been used conventionally for the management of numerous disorders such as rheumatism, cough, headache, fever, tonsillitis, and piles, etc. [10]. *Rumex hastatus* is an annual, biennial and perennial herbs in the *Polygonaceae* family (buckwheat). The plant is a suffrutescent richly branching shrub. It grows up to 90 to 120 cm tall and leaves with petioles of the same length as the blade; blade hastate, panicles terminal with erect divergent, mostly simple branches, nut up to 2 mm long, brown, and long spindle-shaped roots. Plants of the *Polygonaceae* family are commonly perennial herbs that are cultivated in acidic, sour soils mostly in the northern hemisphere. In India, it is extensively dispersed in the Himalayas, Himachal Pradesh, Uttaranchal and Kumaun. In Pakistan, it is present in hilly areas of Batkhela, KPK and Jammu and Kashmir. Roots of *Rumex hastatus* have great value in Ayurveda and other traditional medications for the management of various ailments like rheumatoid arthritis, diarrhea, dysentery, diuretic, wound healing, neoplasm, jaundice, etc. Phytochemical examination of *Rumex hastatus* has shown the occurrence of nepodin, rumexoside, orientaloside, hastatuside B, nepodin-8-O-β-D-glucopyranoside, rutin, vitexin, luteolin and luteolin-7-O-glucoside. Though, medicinal confirmation is not present in writings to validate the statements by folk medicine experts of the beneficial properties of the *Rumex hastatus*. It has been assessed for anti-cholinesterase, anti-oxidant, antitumor, antiangiogenic, phytotoxic and anti-bacterial properties [11,12]. 

Diseases of the gastrointestinal tract (GIT) form a major burden on health. In the Asian population, disorders of GIT are highly prevalent and also play a major role in various comorbidities. GIT disorders have a high rate of prevalence due to lifestyle disturbances, poor hygiene, and increases exposure to infection and other related environmental aspects are also involved [8]. Multiple disorders are involved in GIT as it is expanded in various body organs and covers a larger area. The prevalence of GIT disorders is increasing around the globe. IBS, gastric and peptic ulcer, colorectal cancers, constipation, diarrhea, GERD and *H. pylori* infection are some of the GIT disorders that are prevalent worldwide. Surveys conducted by World Gastroentrology Organization concluded that in the adult population *H. pylori* infection are the most prevalent and shows a 50% median prevalence globally [13]. According to a WHO report on recent global disease burden, diarrhea is one of the top ten diseases that leads to death globally [10].

There are several signal transduction mechanisms (Muscuranic receptor, PGE2, Gastrin and Histamine) involved in the gastric parietal cells, which are linked to the H^+^/K^+^-ATPase. It is catalyzed by the interchange of intracellular H^+^ and extracellular K^+^, as well as cytoplasmic ATP hydrolysis. It is responsible for the crucial factor that causes gastric acid secretion. An imbalance between defensive and offensive agents causes a stomach ulcer, which directly compromises the integrity of the gastric mucosal barrier. Excess gastric acid, on the other hand, is one of the most offensive elements that lead to all types of gastric ulcer illnesses. Literature indicates that ethanol and nonsteroidal anti-inflammatory drugs (NSAIDs), such as aspirin, ibuprofen, and indomethacin, can promote gastric acid secretion, damage the gastric mucosa and disrupt the gastric mucosal barrier. All of the above, however, are linked to H^+^/K^+^-ATPase malfunction [8]. In the clinic, inhibiting the activity of the H^+^/K^+^-ATPase becomes a significant treatment for gastric ulcers. 

A lot of discoveries are made in the field of medicine but there is no ideal treatment for gastrointestinal diseases. There is a lot of room for research in this field of medicine. Moreover, available treatment options provide relief for a shorter time duration, whereas extracts of medicinal plants are considered as a safer therapeutic option and they also improve the GI disorder for a longer duration of time. Natural drug compounds and herbal extracts form a major proportion of drugs available around the globe and are considered as a major part of modern medicine, i.e., 61% [14,15].

This present study aimed to delineate *Rumex hastatus* extracts and their phytoconstituent for antidiarrheal effects, antisecretory effects, isolated tissue preparation, gastrointestinal transient time, anti *H. pylori*, and antiulcer effects. Another aim of this study is to demonstrate whether *Rumex hastatus* exerts a gastroprotective role in ethanol-induced ulcers via the H^+^/K^+^-ATPase-dependent pathway.

## 2. Results

### 2.1. Phytochemical Analysis

**Figure 1:**
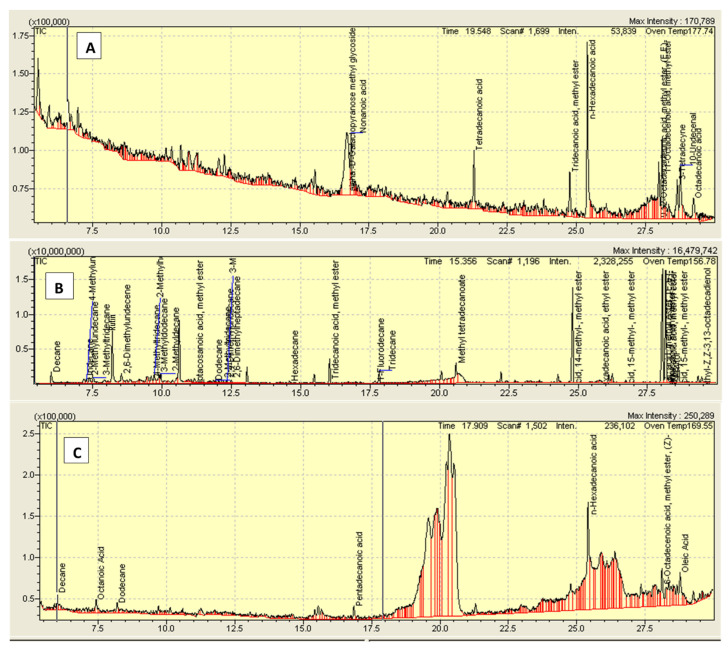
Chromatogram of (**A**) crude methanolic extract of *Rumex hastatus* (Rh.Cr), (**B**) n-hexane (Rh.n-Hex) and (**C**) ethyl acetate fraction (Rh.ETAC).

The phytochemical analysis of *Rumex hastatus* showed different categories of phytoconstituents (Table 1) and shows different peaks in GC-MC chromatogram (Figure 1, Table 2).

### 2.2. Effect on Castor Oil-Induced Diarrhea

In the castor oil-induced diarrheal model, the Rh.Cr, fractions, and rutin significantly prolonged the onset of diarrhea and reduced the frequency and weight of fecal outputs at doses of 50–300 mg/kg as compared to the control. Data revealed that the percent inhibition of defecation for Rh.Cr at the dose of 50, 100, and 300 mg/kg were 41.6, 80.5, and 100%, respectively. Coming to solvent fractions both Rh.n-Hex and Rh.ETAC has significant results. The diarrheal inhibition obtained as compared to control were 45.8, 59.7 and 80.5% at a dose of 50–300 mg/kg Rh.nHex, respectively. At a dose of 50–300 mg/kg the inhibition for Rh.ETAC were 23.6 (* *p* < 0.001), 37.5 and 62.5% respectively. Similarly, rutin has significant results at doses of 50–200 mg/kg, i.e., 41.6, 80.5, and 100%, respectively. Loperamide at dose of 2 mg/kg has 100% inhibition. All of them also showed a significant reduction in weight of both wet and total fecal outputs at all doses. Rh.Aq have weak antidiarrheal effect (Table 3).

### 2.3. Effect on Intestinal Fluid Accumulation

In castor oil-induced intestinal fluid accumulation assay, Rh.Cr, Rh.nHex, Rh.ETAC, Rh.Aq and rutin exhibited a dose-dependent (50–300 mg/kg) anti-secretory effect. Intestinal fluid accumulation in the saline-treated group was 89.7 ± 0.93 (mean ± SEM, *n* = 5), whereas in the castor oil-treated group it was raised to 126.8 ± 0.73. The Fluid accumulation was significantly decreased by Rh.Cr, Rh.nHex, Rh.ETAC, Rh.Aq and rutin at dose of 50–300 mg/kg to 119, 105, 85, 120.6, 110, 92.8, 118, 99, 78, 124, 122, 118, 109, 85 and 75 respectively. Atropine at the dose of 0.1 mg/kg decreased the intestinal fluid accumulation to 74.10 ± 0.42 (Table 4).

### 2.4. Effect on Charcoal Meal Transit Time

Rh.Cr, fractions and rutin delay the charcoal meal to travel through the small intestine in a dose-dependent manner. Percent inhibition in charcoal meal transit by the Rh.Cr, Rh.nHex, Rh.ETAC, Rh.Aq and rutin at 50, 100 and 300 mg/kg dose is 28.83, 48.6, 79.18, 15.4, 26.70, 41.09, 15.40, 54.16, 66.07, 1.44, 6.1, 9.37, 11.94, 32.85 and 74.1 respectively. Atropine (0.1 mg/kg, i.p.) shows inhibitory effect of 81.40% (Table 5).

### 2.5. Effect of Extracts and Rutin on Motility of Isolated Tissue Preparations

**Figure 2:**
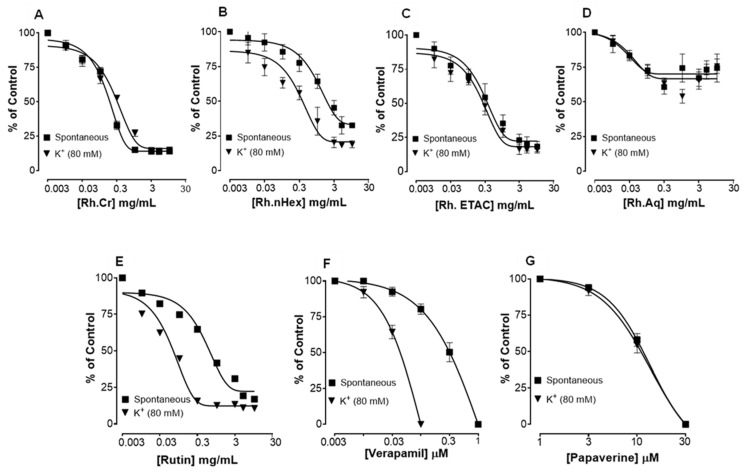
Concentration-dependent inhibitory effect on spontaneous and K^+^ (80 mM) induced contractions of (**A**) *Rumex hastatas* crude extract (Rh.Cr) and fractions: (**B**) n-hexane (Rh.n-Hex), (**C**) ethyl acetate (Rh.ETAC), (**D**) aqueous (Rh.Aq), (**E**) rutin (**F**) verapamil and (**G**) papaverine in isolated rabbit jejunum preparations. Values are expressed as mean ± SEM (*n* = 4–5).

**Figure 3:**
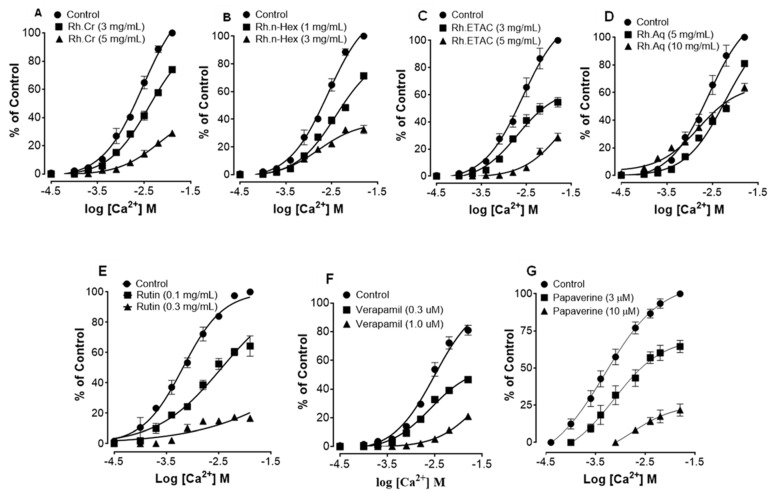
Concentration-response curves of Ca^2+^ in the absence and presence of the increasing concentrations of (**A**) *Rumex hastatas* extract (Rh.Cr) and fractions: (**B**) n-hexane (Rh.n-Hex), (**C**) ethyl acetate (Rh.ETAC), (**D**) aqueous (Rh.Aq), (**E**) rutin (**F**) verapamil and (**G**) papaverine in isolated rabbit jejunum preparations. Values are expressed as mean ± SEM (*n* = 4-5).

**Figure 4:**
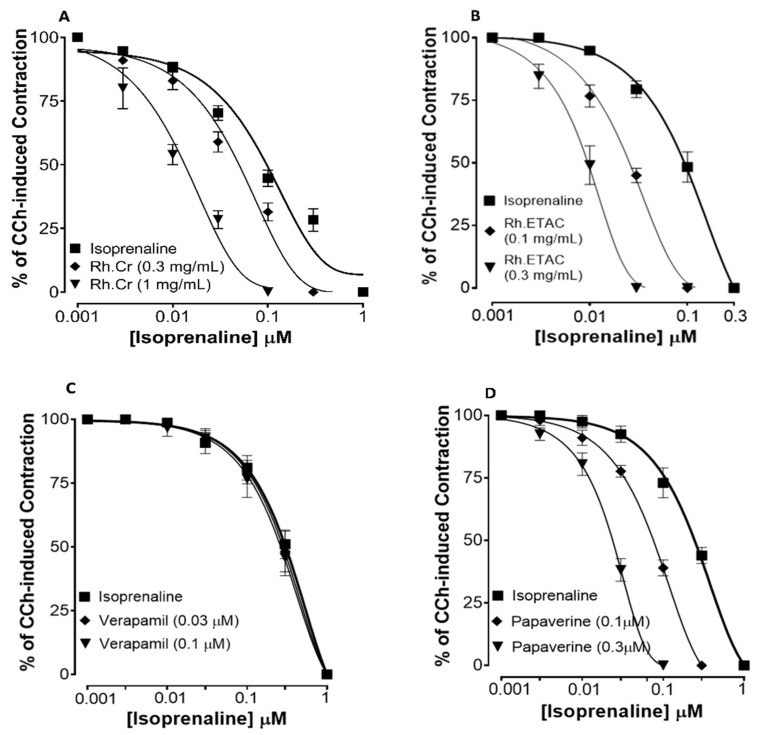
Concentration–response curves of isoprenaline against carbachol (CCh)-induced contractions in the presence of different concentrations of (**A**) *Rumex hastatas* extract crude (Rh.Cr), (**B**) ethyl acetate fraction (Rh.ETAC) (**C**) verapamil and (**D**) papaverine in isolated rabbit jejunum preparations. Values are expressed as mean ± SEM (*n* = 4–5).

Figure 2 shows the inhibitory effect of the plant extract, papaverine, and verapamil against spontaneous and K^+^ (80 mM) induced contractions. Rh.Cr was found to be equally effective against spontaneous and K^+^ (80 mM) induced contractions with EC values of 0.061 mg/mL (0.0187–0.197) and 0.117 (0.031–0.452) respectively, as shown in Figure 2A. Similarly, papaverine also showed a similar pattern of nonspecific inhibitory response (Figure 2G) with respective EC50 values of 0.4 µM (0.22–0.81, *n* = 4) in spontaneous and 0.6 (0.31–1.32, *n* = 4) in high K^+^ (80 mM) induced contractions, whereas verapamil was found more potent against K^+^ (80 mM) induced contractions with EC_50_ value of 0.04 µM (0.031–0.062, *n* = 4), as compared to spontaneous contractions (0.12 µM (0.10–0.20, *n* = 3)), as shown in Figure 2F. Ethyl acetate fraction (Rh.ETAC) produced similar nonspecific inhibitory response as papaverine with EC50 value of 0.246 mg/mL (0.160–0.377) against spontaneous and 0.163 mg/mL (0.0854–0.311) against K^+^ induced contractions Figure 2C, hexane fraction (Rh.nHex) and rutin shows verapamil like response with EC50 values of 1.140 mg/mL (0.682–1.906) and 0.745 mg/mL (0.342–1.623) respectively against spontaneous contractions and 0.197 mg/mL (0.075–0.514) and 0.031 (0.017–0.056) respectively against K^+^ induced contractions Figure 2B,E whereas aqueous fraction (Rh.Aq) shows weak response against both contractions Figure 2D. When tested for its possible interaction with calcium channels, except Rh.Aq, caused a rightward shift in the Ca^2+^ CRCs, similar to that produced by papaverine or verapamil (Figure 3). When tested for possible interaction with isoprenaline, pretreatment of the tissue with Rh.Cr (0.3–1 mg/mL) and Rh.ETAC (0.1–0.3 mg/mL) shifted the isoprenaline-induced inhibitory CRCs to the left, showing potentiating effect. Papaverine also caused a similar concentration-dependent leftward shift in the CRCs of isoprenaline, while pretreatment of tissue with verapamil did not alter the inhibitory response to isoprenaline (Figure 4).

### 2.6. Anti H. pylori Effect

Zone of inhibition and MIC values were assessed against *H. pylori* isolate which showed more sensitivity by disc diffusion method for the two most potent fractions (Rh.n-hex and Rh.ETAC) and phytoconstituent rutin. MIC and zone of inhibition values are shown in Table 6.

### 2.7. Effect on Ethanol-Induced Ulcer

**Figure 5:**
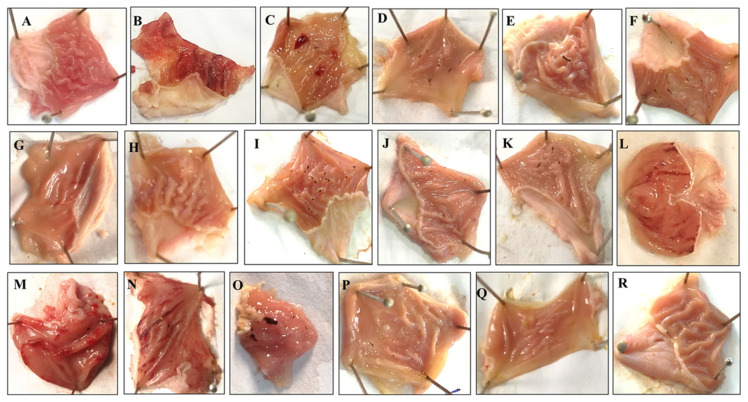
Gross-appearance of gastric mucosa in rats: Pretreated with (**A**) saline (10 mL/kg), (**B**) ethanol (1 mL/100 g) severe injuries are seen, as ethanol produced excessive hemorrhagic necrosis of gastric mucosa, pretreated with (**C**) *Rumex hastatus* crude extract (Rh.Cr 50 mg/kg), (**D**) Rh.Cr 100 mg/kg, (**E**) Rh.Cr 300 mg/kg and fractions: (**F**) n-Hexane (Rh.n-Hex 50 mg/kg), (**G**) Rh.n-Hex 100 mg/kg, (**H**) Rh.n-Hex 300 mg/kg, (**I**) ethyl acetate (Rh.n-ETAC 50 mg/kg), (**J**) Rh.ETAC 100 mg/kg, (**K**) Rh.ETAC 300 mg/kg, (**L**) aqueous (Rh.Aq 50 mg/kg), (**M**) Rh.Aq 100 mg/kg, (**N**) Rh.Aq 300 mg/kg, (**O**) rutin 50 mg/kg, (**P**) rutin 100 mg/kg, (**Q**) rutin 200 mg/kg and (**R**) omeprazole 20 mg/kg.

Rh.Cr, fractions and rutin in a dose-dependent manner (50–300 mg/kg) exhibited an antiulcer effect. Rh.Cr, Rh.nHex, Rh.ETAC, Rh.Aq and rutin at 50, 100 and 300 mg/kg dose caused 30, 87, 92, 47, 76, 100, 35, 52, 72, 0, 2, 5.88, 56.8, 73.4 and 89.4% inhibition, respectively (Table 7). Omeprazole (20 mg/kg) exhibited 96% inhibitory effect. Macroscopic observation showed the gastric mucosa of rats (Figure 5).

### 2.8. H^+^/K^+^-ATPase Inhibition

**Figure 6:**
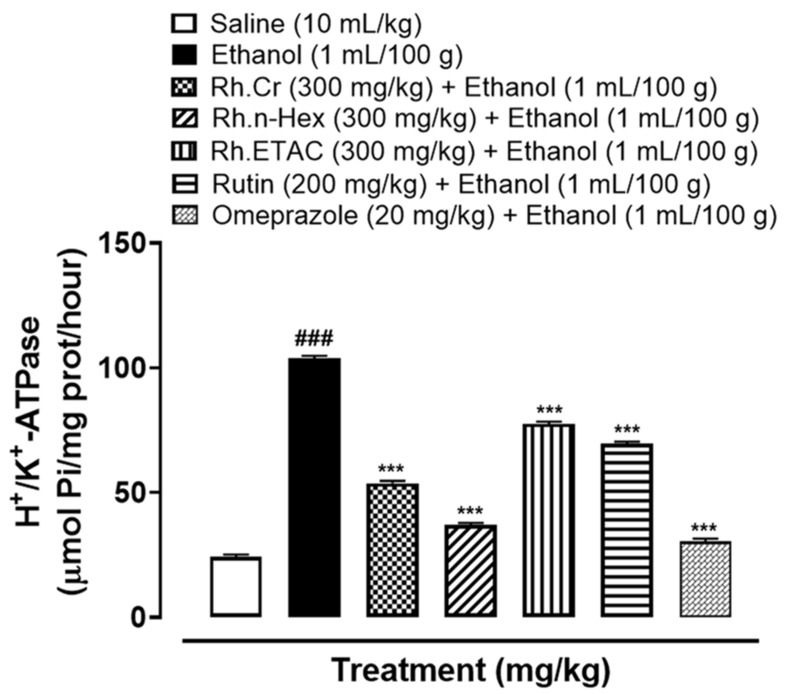
Effect of *Rumex hastatus* crude extract (Rh.Cr), n-hexane (Rh.n-Hex), ethyl acetate (Rh.ETAC) fraction, rutin and omeprazole against H^+^/K^+^-ATPase in ethanol-treated rats gastric tissues. Values are expressed as mean ± SEM (*n* = 3). One-way ANOVA with *post-hoc* Tukey’s test. ^###^ *p* < 0.001 vs. saline group, *** *p <* 0.001 vs. ethanol group.

Abnormal activity of H^+^/K^+^-ATPase is closely related to gastric ulcers. In the ethanol-treated group (1 mL/100 g) the H^+^/K^+^-ATPase activity was significantly increased. Treatment with Rh.Cr, Rh.n-Hex, Rh.ETAC (300 mg/kg), rutin (200 mg/kg) and omeprazole (20 mg/kg) considerably reduced the activity (Figure 6).

### 2.9. Antioxidant Profile

**Figure 7:**
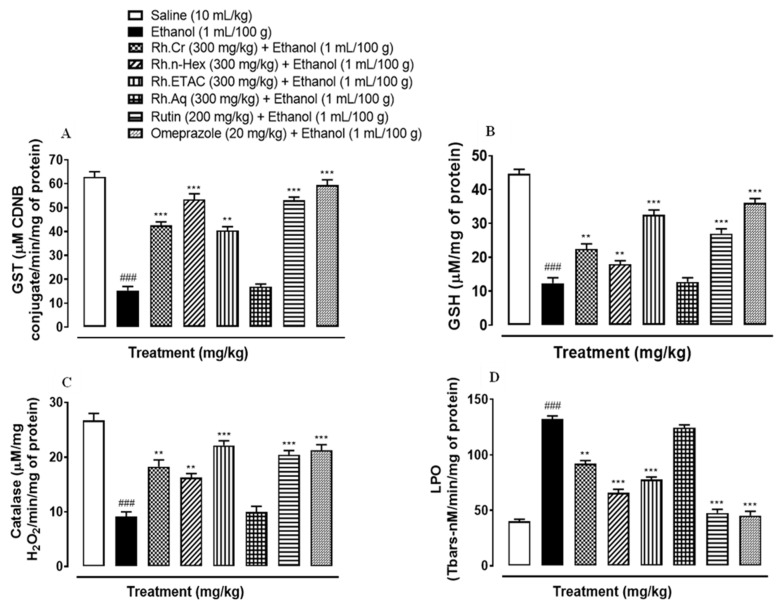
Effect of *Rumex hastatus* crude extract (Rh.Cr), n-hexane (Rh.n-Hex), ethyl acetate (Rh.ETAC), an aqueous fraction (Rh.Aq), rutin and omeprazole groups against (**A**) glutathione-S-transferase (GST), (**B**) reduced glutathione (GSH), (**C**) catalase and (**D**) lipid peroxidation (LPO) in ethanol-treated rats gastric tissues. Values are expressed as mean ± SEM (*n* = 3). One-way ANOVA with *post-hoc* Tukey’s test. ^###^ *p* < 0.001 vs. saline group, ** *p* < 0.01, *** *p* < 0.001 vs. ethanol group.

The activity of GST, GSH and catalase was significantly reduced while that of LPO increased in ethanol induced ulcer gastric tissues. Rh.Cr, Rh.n-Hex, Rh.ETAC (300 mg/kg), rutin (200 mg/kg) and omeprazole (20 mg/kg) treated groups restored GST, GSH, catalase, and considerably reduced LPO. Rh.Aq (300 mg/kg) treated group has a weak effect on oxidative stress markers (Figure 7).

### 2.10. Histopathological Examination

**Figure 8:**
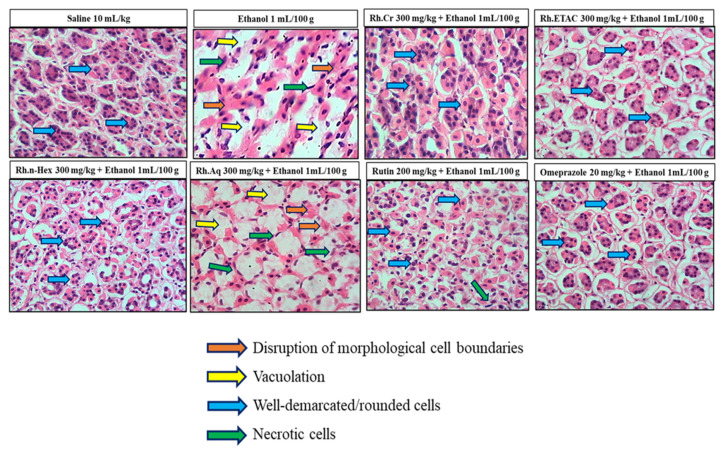
Histopathological slides showing the effect of *Rumex hastatus* crude extract (Rh.Cr), n-hexane (Rh.n-Hex), ethyl acetate (Rh.ETAC), an aqueous fraction (Rh.Aq), rutin and omeprazole in ethanol-treated rats gastric tissues using hematoxylin and eosin staining histopathological technique. Bar 50 µm, magnification 40×.

Figure 8 indicates the H&E staining performed on the gastric tissue. The gastric cells were evaluated for cellular changes or being intact in a normal state. Saline group (10 mL/kg) indicated intact shape, size and stain of the gastric cells, ethanol group (1 mL/100 g) indicates vigorous cellular changes as necrotic cells and hemorrhage can be observed with disruption of the morphological cell boundaries. Comparatively the Rh.Cr, Rh.n-Hex, Rh.ETAC (300 mg/kg), rutin (200 mg/kg) and omeprazole (20 mg/kg) treatment groups revealed relatively intact cell boundaries and cell morphological features as size, shape, vacuolation, and staining. Rh.Aq (300 mg/kg) treated group has relatively weak effects in restoring the cell morphological features.

### 2.11. IHC Analysis

**Figure 9:**
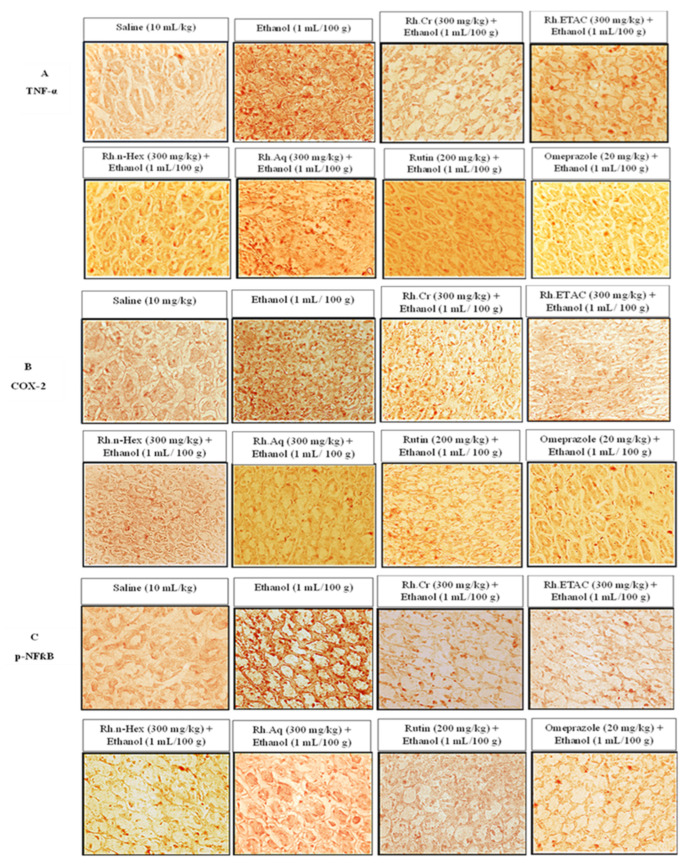
Effect of *Rumex hastatus* crude extract (Rh.Cr), n-hexane (Rh.n-Hex), ethyl acetate (Rh.ETAC), aqueous fraction (Rh.Aq), rutin and omeprazole on apoptotic markers (**A**) Tumor necrosis factor alpha (TNF-*α*), (**B**) cyclooxygenase 2 (COX-2) and (**C**) phosphorylated-nuclear factor kappa B (p-NF*κ*B) in ethanol-treated rats gastric tissues using the immunohistochemical technique. Bar 50 µm, magnification 40×.

**Figure 10:**
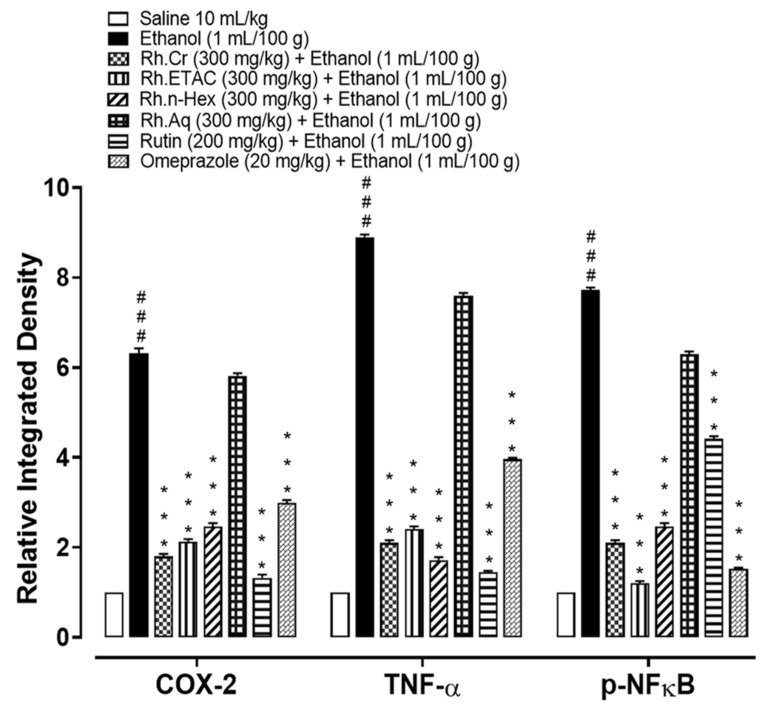
Inhibitory effect of *Rumex hastatus* crude extract (Rh.Cr), n-hexane (Rh.n-Hex), ethyl acetate (Rh.ETAC), an aqueous fraction (Rh.Aq), rutin and omeprazole against cyclooxygenase 2 (COX-2), tumor necrosis factor alpha (TNF-α) and phosphorylated-nuclear factor kappa B (p-NFkB) expression in ethanol-treated rats gastric tissues, using the immunohistochemical technique. Values are expressed as mean ± SEM (*n* = 3). One-way ANOVA with *post-hoc* Tukey’s test. ^###^ *p* < 0.001 vs. saline group, *** *p* < 0.001 vs. ethanol group.

The ethanol (1 mL/100 g) treated group revealed hyper-expression of COX-2, TNFα, and p-NFkB in gastric tissues. In Rh.Cr, Rh.n-Hex, Rh.ETAC (300 mg/kg), rutin (200 mg/kg) and omeprazole (20 mg/kg) treated groups their expression reduced (Figure 9 and Figure 10).

### 2.12. Effect on Inflammatory Markers

**Figure 11:**
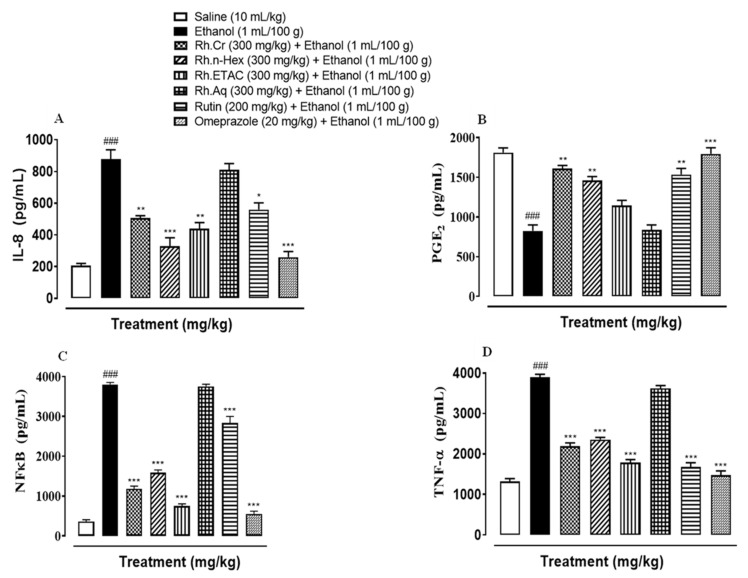
Effect of *Rumex hastatus* crude extract (Rh.Cr), n-hexane (Rh.n-Hex), ethyl acetate (Rh.ETAC), an aqueous fraction (Rh.Aq), rutin and omeprazole groups against (**A**) interleukin 8 (IL-8), (**B**) prostaglandin E2 (PGE2), (**C**) phosphorylated-nuclear factor kappa B (p-NFƙB) and (**D**) tumor necrosis factor alpha (TNF-α) in ethanol-treated rats gastric tissues, measured by enzyme-linked immunosorbent assay (ELISA) technique. Values are expressed as mean ± SEM (*n* = 3). One-way ANOVA with post-hoc Tukey’s test. ^###^ *p* < 0.001 vs. saline group, * *p* < 0.05, ** *p* < 0.01, *** *p* < 0.001 vs. ethanol group.

The levels of IL-8 in the ethanol (1 mL/100 g) treated group were raised to 877.5 pg/mL. The IL-8 expression was significantly decreased in Rh.Cr, Rh.n-Hex, Rh.ETAC (300 mg/kg) and rutin (200 mg/kg) treated groups to 505.5, 328.6, 439 and 550 pg/mL respectively. Omeprazole (20 mg/kg) decreased the expression up to 259.8 pg/mL. Rh.Aq (300 mg/kg) treated group shows weak effect on expression of IL-8, i.e., 795 pg/mL (Figure 11A). The levels of PGE_2_ in the ethanol (1 mL/100 g) treated group was 825 pg/mL. The PGE2 expression was significantly increased in Rh.Cr, Rh.n-Hex, Rh.ETAC (300 mg/kg) and rutin (200 mg/kg) treated groups was found to be 1610, 1457, 1110 and 1535 pg/mL (** *p* < 0.001 versus ethanol group) respectively. Omeprazole (20 mg/kg) raised the levels upto 1780 pg/mL. Rh.Aq (300 mg/kg) treated group did not show any significant results as compared to the control group, i.e., 805 pg/mL (Figure 11B). The levels of p-NFƙB in the ethanol (1 mL/100 g) treated group was 3802.5 pg/mL. p-NFƙB expression was significantly decreased in Rh.Cr, Rh.n-Hex, Rh.ETAC (300 mg/kg) and rutin (200 mg/kg) treated groups to 1180, 1582.5, 752.5 and 2830 pg/mL respectively. Omeprazole (20 mg/kg) decreased the levels to 552.5 pg/mL. p-NFƙB expression in Rh.Aq (300 mg/kg) treated was 3755 pg/mL (Figure 11C). TNFα levels in the ethanol-treated group (1 mL/100 g) were raised to 3896.37 pg/mL. Expression of TNFα was significantly decreased in Rh.Cr, Rh.n-Hex, Rh.ETAC (300 mg/kg) and rutin (200 mg/kg) treated groups to 2185, 2352.5, 1782.5 and 1675 pg/mL respectively. Omeprazole (20 mg/kg) reduced the levels to 1472.67 pg/mL. Rh.Aq (300 mg/kg) treated group shows less effect on TNFα expression, i.e., 3620 pg/mL (Figure 11D).

### 2.13. Quantification of mRNA Levels

**Figure 12:**
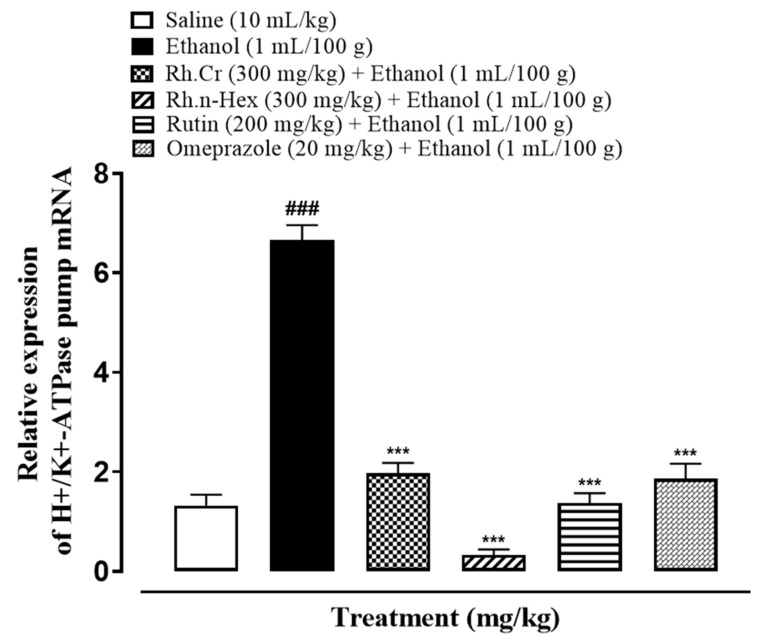
Inhibitory effect of *Rumex hastatus* crude extract (Rh.Cr), n-hexane (Rh.n-Hex) fraction, rutin and omeprazole against mRNA of H^+^/K^+^-ATPase expression in ethanol-treated rats gastric tissues, using Real Time-Polymerase Chain Reaction (RT-PCR) technique. Values are expressed as mean ± SEM (*n* = 3). One-way ANOVA with *post-hoc* Tukey’s test. ^###^ *p <* 0.001 vs. saline group, *** *p <* 0.001 vs. ethanol group.

RT-PCR determined the fold expression of H^+^/K^+^-ATPase in ethanol-induced gastric ulcer model. The ethanol (1 mL/100 g) administered group indicated increased expression of H^+^/K^+^-ATPase mRNA levels. Rh.Cr, Rh.n-Hex (300 mg/kg) and rutin (200 mg/kg) caused a significant decrease in expression levels. Omeprazole (20 mg/kg) also reduced the expression compared to negative control group (Figure 12).

### 2.14. Toxicity Studies

**Figure 13:**
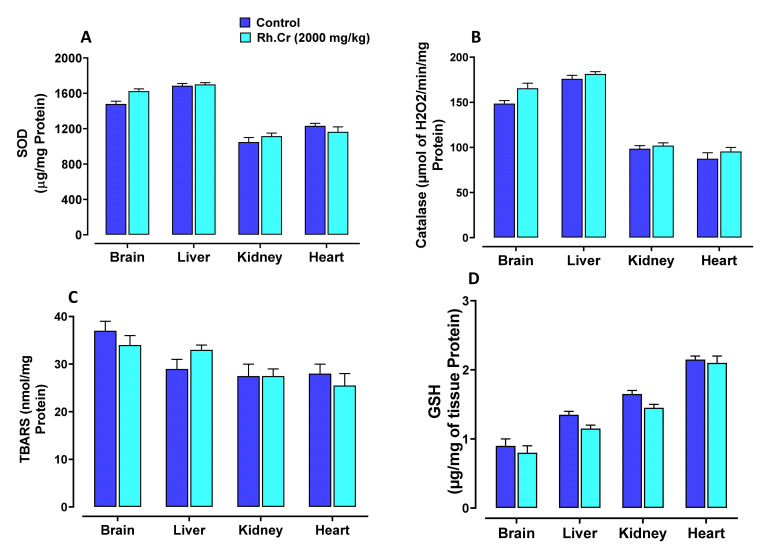
Effect of *Rumex hastatus* crude extract (Rh.Cr) against (**A**) superoxide dismutase (SOD), (**B**) catalase, (**C**) lipid peroxidation (LPO) and (**D**) reduced glutathione (GSH) in different tissues of rats.

**Figure 14:**
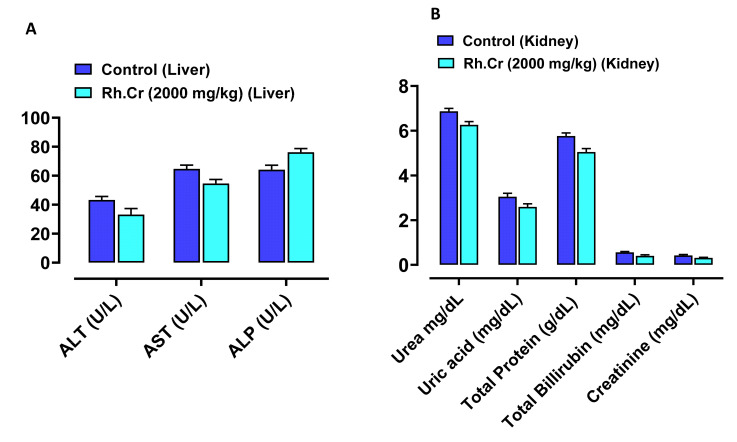
Effect of *Rumex hastatus* crude extract (Rh.Cr) against (**A**) liver function tests (LFTs) ALT (alanine aminotransferase), AST (aspartate aminotransferase) and ALP (alkaline phosphatase) (**B**) renal function tests (RFTs) of rats liver and kidney.

**Figure 15:**
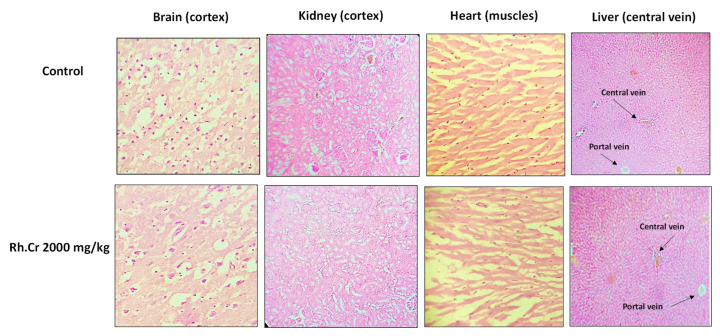
Histopathological slides showing the effect of *Rumex hastatus* crude extract (Rh.Cr) on vital organs of rats using hematoxylin and eosin staining histopathological technique. Bar 50 µm, magnification 40×.

The OECD guidelines 425 were used to evaluate the safety profile of *Rumex hastatus*. After receiving 2000 mg/kg of the extract, fur and skin, fecal consistency, urine color, breathing, and sleep pattern were all normal. There were no symptoms of convulsions or distress in any group of animals. During the 14-day treatment, both groups’ weights progressed normally. On examining the weight of organs no significant variation was found in drug-treated animals as compared to the control group (Table 8). There were no changes in antioxidant profiling, as well as normal LFTs and RFTs. Histopathological examination of important organs such as the brain, liver, kidney, and heart revealed no evidence of vacuolation, dystrophy, or atrophy. *Rumex hastatus* was found to be safe to consume up to a dose of 2000 mg/kg in a comprehensive toxicity profile (Figure 13, Figure 14 and Figure 15).

### 2.15. Molecular Docking

**Figure 16:**
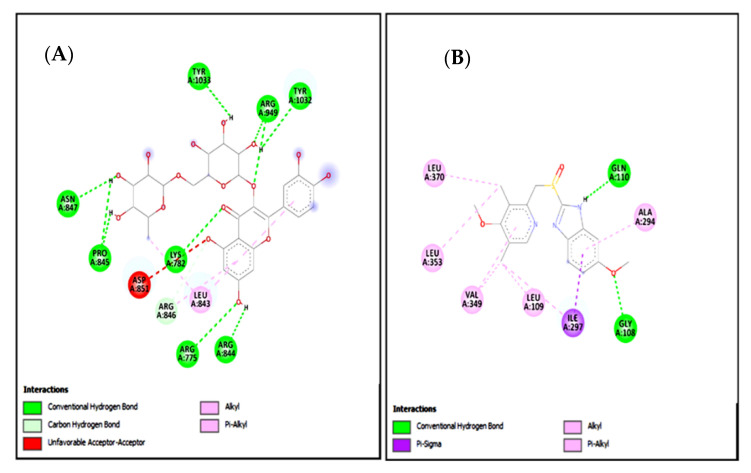
(**A**,**B**) represents 2D interactions of rutin and omeprazole against the target: H^+^/K^+^-ATPase respectively, evaluated through Biovia Discovery Studio 2016.

**Figure 17:**
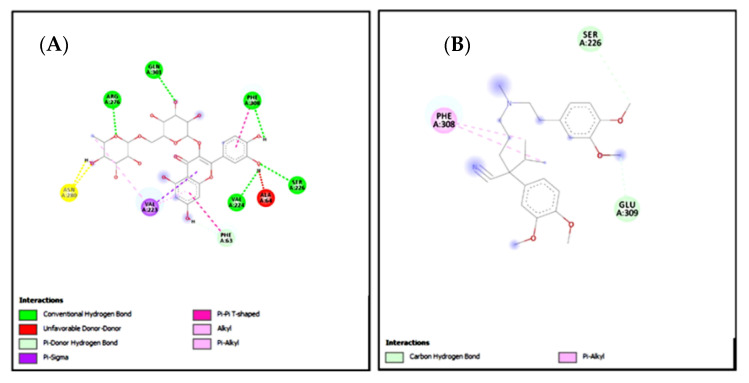
(**A**,**B**) represents 2D interactions of rutin and verapamil against the target: Voltage-gated L-type calcium channels respectively, evaluated through Biovia Discovery Studio 2016.

In the present study, rutin exhibited variable binding affinities against different protein targets. Rutin against H^+^/K^+^-ATPase pump and voltage-gated L-type calcium channel showed E-value of −8.7 and −9.4 Kcal/mol respectively. Omeprazole against the H^+^/K^+^-ATPase pump exhibited an E-value of −7.8 Kcal/mol. Verapamil against voltage-gated L-type calcium channel showed an E-value of −6.2 Kcal/mol. Table 9 shows the atomic energy/E-values (kcal/mol), hydrogen bonding, and residues involved in H-bonding with best-docked poses of the drug-protein complex. Figure 16 and Figure 17 illustrate 2D-view of interactions of rutin and standard drug with their protein targets.

Standard inhibitors of the pathways are omeprazole and verapamil. Amino acids are: alanine (ALA), aspartic acid (ASP), asparagine (ASN), arginine (ARG), cysteine (CYS), glycine (GLY), glutamine (GLN), glutamic acid (GLU), histidine (HIS), isoleucine (ILE), leucine (LEU), lysine (LYS), proline (PRO), phenylalanine (PHE), serine (SER), tyrosine (TYR), tryptophan (TRP), threonine (THR), valine (VAL).

### 2.16. Molecular Dynamic Simulations

**Figure 18:**
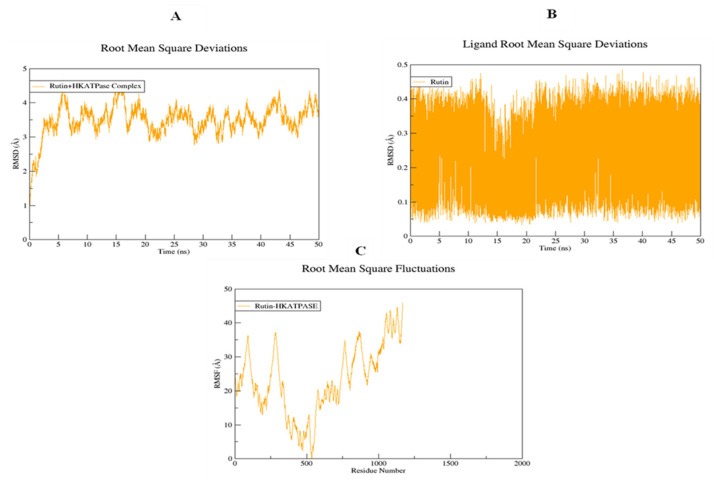
Root mean square deviations of backbone atoms of (**A**) rutin-H^+^/K^+^-ATPase docked complex, (**B**) rutin, and (**C**) root mean square fluctuations of the backbone atoms of the rutin-H^+^/K^+^-ATPase docked complex.

**Figure 19:**
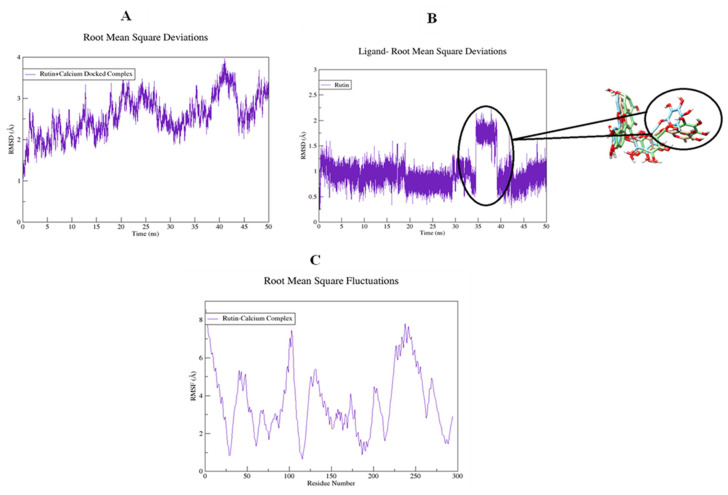
Root mean square deviations of backbone atoms of (**A**) rutin-voltage gated L-type calcium channel docked complex, (**B**) rutin, and (**C**) root mean square fluctuations of the backbone atoms of the rutin-voltage gated L-type calcium channel docked complex.

The structural flexibility of targeted proteins and the stability of docked complexes were investigated using molecular dynamics (MD) simulation. The selected best-docked poses for the complexes were used to initiate the simulation process for the period of 50 ns. The dynamic stabilities of the systems were calculated and plotted using root mean square deviation analysis. For the rutin-H^+^/K^+^-ATPase top-ranked docked complex, trajectories analysis using an RMSD plot confirms the stability of protein backbone atoms throughout the simulation run (50 ns) with an average RMSD value of (3.50 Å) Figure 18A. The rutin trajectory analysis reveals a nearly constant RMSD throughout the simulation process. A constant ligand RMSD, with an average RMSD of 0.23 Å, indicates that the ligand did not flip or move during simulations (Figure 18B). The restriction in the free rotation could be due to the active site’s rigid packing. The RMSF plot revealed that the residues involved in binding were more stable than the rest of the protein’s residues (18C). The average RMSF value was estimated to be 23.06 Å which also highlighted the high degree of flexibility of protein due to loop regions. The average RMSF value of 23.06 Å also indicated that protein was very flexible due to loop sections (Figure 18C). For the rutin-voltage gated L-type calcium channel docked complex, an unobvious pattern of fluctuations was observed both for the backbone atoms of protein as well as for the ligand (Figure 19A,B). The system reached equilibrium after 1 ns and remained stable until 35 ns, with an average RMSD of 2.93 Å. At 35 ns, there is a significant deviation from the initial, resulting in a displacement of 4.00 Å. The same trend was observed when the ligand’s RMSD plot was visualized. To determine the possible cause, snapshots at 32, 36, and 40 ns were extracted and superimposed (Figure 19B). The superimposition of binding conformations from the selected snapshots revealed that the toluene group of rutin moved from its initial orientation at 36 ns of simulation time and returned back to the original conformation at 40 ns. It is possible that this movement was made to fit perfectly within the protein pocket. Overall, the average RMSDs estimated for both the backbone atoms (2.59 Å) and ligand (1.00 Å) were within the acceptable range and showed a normal trend. Aside from conformational drift, the flexibility of the backbone atoms was evaluated by observing how much they varied over the 50 ns of simulation. As shown in Figure 19C, some regions of protein have produced obvious changes. Three major peaks with a high degree of fluctuations were observed, whereas the ligand binding regions showed fewer fluctuations with an average RMSF value of 3.66 Å Figure 19C. The acceptable RMSF value reflected a minor structural rearrangement in the backbone atoms as a result of the movement of the rutin toluene group during the simulation run. The ligand was rescored based on free energies calculated using MMPBSA/GBSA methods for both proteins. All of the energy values were calculated as the average of 150 snapshots from the MD trajectories of the last 10 ns. The total free energies (ΔTOTAL) calculated for rutin + H^+^/K^+^-ATPase docked complex in the case of MMGBSA and MMPBSA were −50.3696 kcalmol^−1^ and −36.8304 kcalmol^−1^ respectively (see Supplementary material, Table S1). On the other hand, the (Molecular Mechanics Generalized Born surface area and molecular mechanics Poisson Boltzmann surface area) MMGBSA and MMPBSA values estimated for rutin + voltage-gated L-type calcium channel were −49.9194 kcalmol^−1^ and −37.5595 kcalmol^−1^, respectively (Table S2). The overall estimated energy values were found to be within an acceptable range and confirming the docked complexes’ stability. Overall the MD simulations MMPBSA/GBSA findings are consistent with the in-vitro, in-vivo and docking results.

## 3. Discussion

Based on *Rumex hastatus* ethnopharmacological use in gastrointestinal problems such as diarrhea, constipation, and gastritis. The antidiarrheal, antisecretory, antispasmodic, charcoal meal gastrointestinal motility, and antiulcer effects of *Rumex hastatus* crude extract, fractions, and one of the primary phytoconstituents were studied. To rationalize the plant’s aforementioned ethnomedicinal uses, in-silico, in-vitro, in-vivo, and molecular approaches were used to elucidate possible underlying mechanism(s).

Alkaloids, anthraquinones, cardiac glycosides, coumarins, flavonoids, saponins, tannins, and terpenoids are among the phytoconstituents found in *Rumex hastatus* extract. GC-MS analysis revealed the presence of different compound peaks. Amongst compound peaks, oleic acid, phthalic acid, octanoic acid, 6-octadecenoic acid, certain glycosides polysaccharides, and other compounds obtained are reported to have effectiveness as anti-inflammatory, anti-ulcer, and in other gastrointestinal diseases [16,17]. The obtained effectiveness in GI ailments can be attributed to the phytochemical profile of this plant. Spectral analysis of *Rumex hastatus* in literature reveals the presence of rutin as one of the main phytoconstituents. HPLC and LCMS data indicating the presence of rutin are available [11,12]. Rutin has been examined for its effectiveness in gastrointestinal ailments. Therefore, we examined different fractions of *Rumex hastatus* along with rutin for its pharmacological efficacy in GIT up to molecular level. 

Rh.Cr, Rh.ETAC, Rh.n-Hex, and rutin showed protective effects against castor oil-induced diarrhea, similar to loperamide, a standard medicine, and its likely underlying mechanism was determined using isolated tissue preparations that were also linked to a reduction in gastrointestinal motility. Castor oil, through its active metabolite, ricinoleic acid, is responsible for raising intestinal fluid and causing diarrhea. It alters electrolyte and water transport, as well as causing massive contractions in the transverse and distal colon [8,18]. In mice, these extracts were found to protect against castor oil-induced intestinal fluid secretion. Antidiarrheal and antisecretory actions are mediated by a gut relaxant constituent found in *Rumex hastatus.* Rh.Aq fraction on the other hand did not show potent antidiarrheal and antisecretory effects.

In test doses Rh.Cr, Rh.ETAC and rutin in the small intestine suppress the propulsion of charcoal marker, similar to the standard medicine atropine sulphate [19], which has a strong anticholinergic activity on intestinal transit. A decrease in GIT motility tone increases the retention of substances in the intestine, allowing for improved water absorption. Rh.n-Hex did not show much potent effect. These findings suggested that the plant had an antimotility effect by altering the peristaltic movement of the gut.

The importance of many physiological mediators, such as acetylcholine, histamine, substance P-cholecystokinins, prostaglandins, and 5-hydroxytryptamine, as well as some ion channels, such as K^+^ or Ca^2+^, in gastrointestinal system regulation is well-recognized. Furthermore, most spasmolytic drugs have been shown to have therapeutic potential in diarrhea by relaxing the smooth muscle of the gut, which helps to keep luminal fluid in the bowl [20,21]. Most plant and plant-based test materials have been found to have inhibitory effects via K^+^ channel activation or Ca^2+^ channel blockade-like mechanisms. Low K^+^ (25 mM) and high K^+^ (80 mM)-induced depolarization in tissues is commonly used to distinguish K^+^ channel opening and Ca^2+^ channel blocking-like activities [22]. K^+^ channel openers and Ca^++^ antagonists cause smooth muscle relaxation by lowering intracellular free Ca^2+^ through respective processes of membrane hyperpolarization, based on the existence of K^+^ channels and voltage-dependent Ca^2+^ channels in intestinal smooth muscles and epithelial cells [23].

In spontaneously contracting rabbit jejunum preparations, the extracts were examined for their probable spasmolytic effect, Rh.Cr and Rh.ETAC suppressed both spontaneous and K^+^-induced contractions with similar potency. Similarly, papaverine, a PDE and Ca^++^ influx inhibitor [8], produced a similar pattern of inhibition with comparable potency against spontaneous and K^+^-induced contractions, whereas Rh.n-Hex and rutin show verapamil like effect, a standard Ca^2+^ antagonist [18], was relatively selective in its inhibitory effect on K^+^-induced contractions. Pretreatment of the tissue with the extracts and rutin shifted the Ca^2+^ CRCs to the right, similar to that generated by papaverine or verapamil, indicating the presence of calcium antagonist constituent(s). The fact that plant extract has a comparable inhibitory pattern to papaverine against spontaneous and K^+^-induced contractions suggests that it may have another mechanism implicated in the spasmolytic effect, such as PDE inhibition. The PDE inhibitory-like effect was confirmed when the Rh.Cr and Rh.ETAC potentiated the isoprenaline-induced relaxant effect, which was similar to that produced by papaverine, whereas Rh.n-hex, Rh.Aq and verapamil had no such effect. 

In the stomach, the H^+^/K^+^-ATPase is found in gastric membrane vesicles and is catalysed by the interchange of intracellular H^+^ and extracellular K^+^, as well as cytoplasmic ATP hydrolysis. It is responsible for the crucial factor that causes gastric acid secretion. An imbalance between defensive and offensive agents causes a stomach ulcer, which directly compromises the integrity of the gastric mucosal barrier. Excess gastric acid, on the other hand, is one of the most offensive elements that lead to all types of gastric ulcer illnesses. Literature indicates that ethanol and nonsteroidal anti-inflammatory drugs (NSAIDs), such as aspirin, ibuprofen, and indomethacin, can promote gastric acid secretion, damage the gastric mucosa, and disrupt the gastric mucosal barrier. All of the above, however, are linked to H^+^/K^+^-ATPase malfunction [24,25]. In the clinic, inhibiting the activity of the H^+^/K^+^-ATPase becomes a significant treatment for gastric ulcers. Rh.Cr, Rh.ETAC, Rh.n-Hex, and rutin are being analyzed for an in-vitro H^+^/K^+^-ATPase inhibitory assay, Rh.n-Hex, and Rh.Cr shows proton pump inhibitory action equivalent to that of the standard medicine omeprazole, an irreversible proton pump inhibitor. RT-PCR study revealed that the expression levels of H^+^/K^+^-ATPase in Rh.n-Hex, Rh.Cr and rutin were dramatically reduced, indicating that the anti-ulcer mechanism of the plant is displayed through the proton pump inhibition pathway at the molecular level. As a result, RT-PCR research confirmed that *Rumex hastatus* exerts its gastroprotective action through a proton pump inhibitory mechanism.

Various aggressive (acid, pepsin, and *Helicobacter pylori* infection) and protective (mucin secretion, prostaglandin, epidermal growth factors, and bicarbonate) factors play a key role in the production and release of acids in the gastrointestinal tract. Disturbance in these variables causes the mucosal barrier to break down, exposing the gastric lining to various enzyme and acid productions, resulting in ulcers [8]. The plant’s beneficial effect was investigated using an ethanol-induced gastric model, which stimulated ulcers through a variety of mechanisms such as free radicals OH, NO production, mucus exhaustion, mucosal damage, and the release of superoxide anion, which ultimately prolonged tissue oxidative stress and numerous studies have suggested that pro-inflammatory mediators such as interleukin-8 (IL-8), TNF-α as well as COX-2 upregulation and p-NF-ƙB activation, play a role in inflammatory cascades [24,26].

The etiology of gastric ulcers is complicated by oxidative stress [27]. Rh.Cr, Rh.ETAC, Rh.n-Hex, and rutin reduced gastric lipid peroxidation and increased GSH, GST, and catalase levels, implying that the plant anti-ulcerogenic properties are linked to its antioxidant profile. The antiulcer activity of extract may refer to its mechanism similar to CCB, as Ca^2+^ antagonist is known for such effects as explored earlier [16].

Monitoring inflammatory mediators may potentially be a useful tool for preventing gastric lesions [1,28]. The protein expression of IL-8, TNF-α, PGE-2, and p-NF-kB was measured using an ELISA method. Rh.Cr, Rh.ETAC, Rh.n-Hex, and rutin cause a considerable reduction in the expression of IL-8, TNF-α, p-NF-ƙB and increased expression of PGE2 in the treated groups. Hence, the plant’s protective action could be attributable to its anti-inflammatory properties. These results are further supported by immunohistochemistry screening of gastric tissues. By lowering the expression of the inflammatory markers COX-2, TNF-α, and p-NF-KB and enhancing the expression of PGE2, the plant protects the gastric tissues. Finally, histological analysis of gastric tissues revealed that the extracts-treated groups had improved cellular infiltration and cell morphology.

Dealing with multidrug-resistant (MDR) microorganisms is a key issue in the chemotherapeutic management of infectious diseases, thus researchers are focused on natural products to discover novel antibacterial, antifungal, and anti-parasitic medicines. Plant-based medicines are a rich source of safe and effective treatments that have been employed in crude form as well as pure isolated substances throughout history [29,30]. We used the disc diffusion method to test the antibacterial potential of *Rumex hastatus* against *H. pylori* in this investigation. The results revealed that Rh.nHex, Rh.ETAC and rutin have substantial antibacterial potential, however, the effect of the remaining fractions was insignificant. The above-mentioned substances’ potent antibacterial effect was also confirmed by determining their MIC. *H. pylori* is the most common cause of peptic ulcers, which are caused by erosion of the gastric and duodenal mucosa.

R.Aq on the other hand did not show promising results in the aforementioned activities that can be justified by the absence of the active phytoconstituent required for effectiveness in GI diseases. Therefore, due to being in an active fraction it was not evaluated further for detailed molecular studies.

To ensure the safety of herbal drugs, a preliminary toxicological examination is required. As a result, the current study was carried out in an animal model to assess the acute toxicity of *Rumex hastatus* crude extract, in accordance with OECD guidelines 425 [31,32], it exhibited a relative safety profile as no impairment was observed in kidneys, heart, liver, and brain further assisted by biochemical analysis.

Molecular docking was used to evaluate the ligand’s affinity for calcium channels and H^+^/K^+^-ATPase. Docking is now used as a preliminary step to confirm the interaction of a ligand with its target [33,34]. The positive docking results in the form of binding affinities and hydrogen bonds were the reason for molecular dynamics (MD) simulations to confirm the interaction of rutin with calcium channels and H^+^/K^+^-ATPase.

Researchers working on drug development and discovery are becoming more interested in MD simulations. This technique can be used to assess conformational changes, atomic structure positioning, identify the mutation, protonation, phosphorylation, and most importantly, the interactions of any atom, target, or ligand with its environment [35]. The MD simulation was initially used in 1970, and it later attracted a large number of scientists, who use it to test their newly developed/discovered compounds. Many researchers have used it to compare and correlate results from animal studies [30]. We also ran 50 ns MD simulations of two complexes. Complexes were evaluated for stability using the Amber 18 software package for 50 nanoseconds. The complexes were found to be stable, with RMSF and RMSD values that were within a range and a favorable interaction that determined the ligands’ affinity for their targets.

In the future, we can target other relevant pathways like histamine, muscarinic, and gastrin receptors which play a crucial role in ulcer pathogenesis. One of the significant limitations of our study is to perform pharmacokinetic studies.

## 4. Materials and Methods

### 4.1. Chemicals

Castor oil was acquired from KCL Pharma, Karachi, Pakistan. Acetylcholine, ethyl acetate, activated charcoal, omeprazole, ethanol, loperamide, papaverine, methanol, n-Hexane, atropine sulphate, potassium chloride, isoprenaline and verapamil hydrochloride (Sigma Chemicals Co, St Louis, MO, USA) were used. 

### 4.2. Animals

Experiments were performed in compilation with rules of the Research and Ethics Committee of Riphah Institute of Pharmaceutical Sciences (Ref. no. REC/RIPS/2019/14) along with the guidelines of “Principles of Laboratory Animal care”. Rabbits (1.0–2.0 kg) albino mice (20–25 g), and Sprague-Dawley rats (150–250 g) of either gender were used in the experimentation procedure, housed in the animal house of the Riphah Institute of Pharmaceutical Sciences (RIPS) Islamabad provided with a controlled environment (20–25 °C).

### 4.3. Plant Material and Phytochemical Screening

The *Rumex hastatus* plant was collected from the hilly area of Batkhela, KPK, Pakistan. After collection plant was authenticated by Dr. Mushtaq Ahmad, a taxonomist at the Department of Plant Sciences, Quaid-a-Azam University Islamabad, and the voucher/specimen number (ISL-B-24) was collected after submitting the sample the specimen to the herbarium at same Department. The plant was dried under shade. The dried materials were powdered and then soaked in aqueous methanol (70% methanol) for 15 days at room temperature. The hydro methanolic extract (550 g) was collected and filtered. This process was repeated 2 more times and under reduced pressure, the filtrates were dried through a rotary evaporator. Semi-solid mass obtained was further processed for fractionation in different solvents according to their polarity order (n-Hexane—Ethyl acetate—Methanol—Aqueous). Fractions of (n-Hex) n-hexane (60 g), (ETAC) ethyl acetate (150 g) and (Aq) aqueous (55 g) were obtained and stored for analysis.

The phytochemical screening was performed to determine the phytoconstituents present in the crude extract and all the fractions of *Rumex hastatus* by following the protocols reported in the literature [36].

### 4.4. Gas Chromatography Mass Spectrometry (GC-MS) Analysis 

A GC-MS (GC-MS QP-2010 Plus Shimadzu, Japan) with a DB-5MS capillary column (30 m × 0.25 mm I.D, 0.25 μm film thicknesses, Shimadzu, Japan) was used. The inlet temperature was maintained at 250 °C. The oven temperature was initially at 50 °C, programmed to 220 °C at 5 °C/min and then programmed to 300 °C at 10 °C/min holding for 15 min. Helium was used as carrier gas at a constant flow rate of 1.0 mL/min. Injection volume is 1 μL. The samples were analyzed by GC–MS with the pulsed splitless injection mode. The ion source was set to 280 °C and the MS transfer line was set to 280 °C. Ionization was carried out in electron impact ionization (EI) mode at 70 eV. The mass spectra were recorded within 50–500 amu in full scan mode to collect the total ion current (TIC).

### 4.5. Castor-Oil Induced Diarrhea

This protocol was carried out according to the instructions provided by [37]. Before the experiment, mice were randomly assigned to one of 17 groups (*n* = 5) and fasted for 24 h (08:00–08:00). Animals were kept in separate cages with absorbent paper lining the floor. Loperamide hydrochloride (2 mg/kg) was given to the positive control group, while the negative control group was given an equal volume of saline (10 mL/kg) and after 1 h of treatment, mice were given castor oil (10 mL/kg p.o.). Diarrhea was induced with castor oil (10 mL/kg, p.o.) after administration of 50, 100 and 300 mg/kg doses of Rh.Cr, Rh.nHex, Rh.ETAC, Rh.Aq and rutin, respectively. The animals were separated into white paper-lined cages. The onset of diarrhea, frequency of defecation, and weight of fecal output (wet and total feces in gram) for each mouse were recorded throughout a 4-h period. The percentages of diarrheal inhibition and fecal output weight were calculated using Formulas (1)–(3).

% of inhibition = Average number of WFC − Average number of WFT/Average number of WFC × 100, where, WFC = wet feces in the control; WFT = wet feces in the test group(1)

Percentage of wet fecal output/WWFO = Mean weight of wet feces of each group/Mean weight of wet feces of control × 100(2)

Percentage of total fecal output/WTFO = Mean weight of total feces of each group/Mean weight of total feces of control × 100(3)

### 4.6. Assessment of Intestinal Fluid Accumulation

The method described by [8] was used to determine the amount of fluid in the intestine. Mice that had been fasted for 24 h (08:00–08:00) were separated into 18 cages, each with five mice. Normal saline (10 mL/kg) and castor oil (10 mL/kg, p.o.) were given to groups I and II, respectively. The remaining groups received 50, 100, and 300 mg/kg intraperitoneally of Rh.Cr, Rh.nHex, Rh.ETAC, Rh.Aq and rutin, respectively 1 h before castor oil. A group was given the standard drug atropine at a dose of 0.1 mg/kg one hour before induction with castor oil (10 mL/kg, p.o.). After 30 min, the mice were sacrificed and their intestines were removed and weighed. The results were written as (Pi/Pm) × 1000, where Pi is the intestine’s weight (g) and Pm is the animal’s weight.

### 4.7. Charcoal Meal Transit Time

A charcoal meal inhibitory activity in rats was calculated (37). The rats were fasted for 24 h and were separated into 18 cages each with five rats, but were allowed to drink freely. Rh.Cr, Rh.nHex, Rh.ETAC, Rh.Aq and rutin were given in doses of 50, 100, and 300 mg/kg body weight in the test groups, while atropine sulphate (0.1 mg/kg, i.p.) was given to the positive control group. The negative control group received normal saline (10 mL/kg, p.o.) and 1 h after the pre-treatment period marker (25 mg/kg) (10 percent charcoal suspension in 5 percent gum acacia) was given to all groups. Animals were sacrificed for 30 min after all treatments. The small intestine was removed and the distance traveled by charcoal meal through the organ was calculated as a percentage of the small intestine’s length using the formula below.

Peristaltic index (PI%) = (Distance moved by charcoal meal/total length of intestine) (cm) × 100

For further evaluation of % inhibition, the peristaltic index is used.

% inhibition = (PIC − PIT)/PIC × 100

where, PIC = peristaltic index of control; PIT = peristaltic index of test group.

### 4.8. Effect of Extracts and Rutin on Motility of Isolated Tissue Preparations

The rabbits (15 rabbits) were fasted for 24 h (08:00–08:00) before the experiment but had free access to water. The jejunal section was isolated and cleaned with Tyrode’s solution after cervical dislocation, about 10–12 cm after the stomach. After the instrument was calibrated, a 2 cm long jejunal segment was placed in a tissue bath containing Tyrode’s solution for 30 min to equilibrate with the environment while having a proper supply of oxygen (95 percent O_2_) and 5 percent CO_2_ (carbogen). A 0.3 M concentration of ACh was used to stabilize each preparation. A force-displacement transducer (model FT-03) was used in conjunction with a bridge amplifier and power Lab 4/25 data collection equipment connected to a computer running Lab-Chart 6 software to collect responses (AD Instrument, Sydney, Australia). The extracts were examined for percent change in jejunum contractions at various concentrations [38]. To determine the calcium channel blocking activity, high K^+^ (80 mM) depolarizes the preparations for smooth muscle contractions by opening voltage-dependent Ca^2+^ channels, allowing extracellular Ca^2+^ to influx, resulting in a contractile effect, and a substance that inhibits high K^+^ induced contraction is considered a blocker of Ca^2+^ influx through L-type Ca^2+^ channels. After the generated contraction had reached a plateau (typically within 7–10 min), test dosages were added in a cumulative manner to get concentration-dependent inhibitory responses. The tissue was allowed to stabilize in normal Tyrode’s solution for 30 min before being replaced with Ca^2+^-free Tyrode’s solution containing EDTA (0.1 mM) to validate the test substance’s Ca^2+^ antagonist effect. Furthermore, tissue was immersed in a K^+^-rich, Ca^2+^-free Tyrode’s solution with the following concentrations (mM): NaCl 91.03, NaHCO_3_ 11.9, NaH_2_PO_4.2_H_2_O 0.32, EDTA-Na_2_·2H_2_O 0.1, KCl 50, MgCl_2_·6H_2_O 0.50, and glucose 5.05. Control concentration-response curves (CRCs) of Ca^2+^ were produced after a 30-min incubation period. The tissue was pretreated with a test dose for 1 h after the control Ca^2+^ CRCs were confirmed super-imposable (typically after two cycles). The potential PDE inhibitory effect was investigated indirectly by constructing isoprenaline-induced inhibitory CRCs against CCh-induced contractions in the absence and presence of the plant extract [39].

### 4.9. Anti-Helicobactor pylori (H. pylori) Activity

The *H. pylori* clinical isolates with resistance profiles were obtained from Breath MAT Lab., Pakistan Institute of Nuclear Science and Technology, Islamabad, Pakistan. They were identified by microaerophilic growth (at 37 °C), colony morphology, Gram staining, catalase oxidase, and urease tests. Antibacterial effect of Rh.Cr, Rh.nHex, Rh.ETAC, Rh.Aq and rutin were analyzed by disc diffusion method, measuring the zone of inhibition in mm using 5 mg of extract per disc. The *H. pylori* clinical isolate SJ2013 10^8^ cfu/mL resistant to commonly used antibiotics such as metronidazole, clarithromycin, and amoxycillin were inoculated on Columbia blood agar (CM 0331B, Oxoid, UK) enriched with 5% defibrinated sheep blood. The dried extract-impregnated discs were applied and incubated at 37 °C for 72 h under microaerophilic conditions by using a gas generating kit, i.e., Campylobacter gas generating kit, (BR 0056A, Oxoid, UK). After 72 h, the zones of inhibition were measured by determining their diameters. The concentrations of minimum inhibitory (MIC) were assessed for the most potent extracts and compounds by microdilution using brain heart infusion (BHI) broth. The extracts and compounds were serially diluted by two folds in BHI broth with serum. The final concentrations of extracts were 0.625 to 5.0 mg/mL. The concentration with no visual growth or turbidity was considered MIC (33).

### 4.10. Ethanol-Induced Ulcer

Rats weighing (250–280 g) of either sex were randomly allocated to 18 groups (*n* = 5) and fasted for 24 h (09:00–09:00). Group 1 was given normal saline as a negative control (10 mL/kg of body weight), remaining groups received 50, 100 and 300 mg/kg, (p.o.) of Rh.Cr, Rh.nHex, Rh.ETAC, Rh.Aq and rutin. The last group received 20 mg/kg, (p.o.) omeprazole as a standard drug. After 1 h of treatment, all of the animals were given 1 mL/100 g of ethanol (p.o.) to produce a gastric ulcer. One hour after ethanol treatment, animals were sacrificed by cervical dislocation. The stomachs were removed and cleansed in normal saline before the lesion index was calculated by measuring each lesion in millimeters along its larger curvature. Each lesion’s surface area was measured and a score was assigned [40,41]. The ulcer index was calculated as the mean ulcer score for each gastrointestinal lesion (US) The ulcer index was calculated by adding the lengths (mm) of all lesions for each gastric injury (UI). The gastroprotective evaluation was expressed as an inhibition percentage (I%), which was calculated using the formula:

$$\%\;\mathrm{Inhibition}=\frac{\left(\mathrm{USc}-\mathrm{USt}\right)}{\mathrm{USc}}\times 100$$

where USc = ulcer surface area of control and USt = ulcer surface area of test drug group. 

For additional proteomic screening, the stomach tissues were preserved in a biofreezer (−80 °C).

### 4.11. H^+^/K^+^-ATPase Inhibitory Activity

The inhibitory effect of extracts on rat gastric H^+^/K^+^-ATPase was analyzed by using the colorimetric method. The commercially available colorimetric H^+^/K^+^-ATPase activity assay screening kit (catalog No E-BC-K122-S, Elabscience, Houston, Texas, USA) was used for analysis. Stomach tissues kept at biofreezer (−80 °C) for storage were homogenized at 15,000 using SilentCrusher M (Heidolph, Schwabach, Germany), the homogenate was then centrifuged at 3500 rpm for 10 min and the supernatant was collected. The resulting supernatant was analyzed for the release of inorganic phosphate after ATP hydrolysis spectrophotometrically at 660 nm. One ATPase activity unit is defined as 1 µmol of inorganic phosphorus released by ATP hydrolysis by ATPase of 1 mg of tissue protein per hour and results were expressed as µmol Pi/mg prot/hour [24].

### 4.12. Determination of Oxidative Stress Markers

The isolated rat gastric tissues were homogenized and centrifuged at 1500 rpm for 30 min to separate the supernatant. The supernatants were analyzed for glutathione (GSH), glutathione-S-transferase (GST), catalase, and lipid peroxidation (LPO) levels. The GSH level was determined by oxidizing GSH and DTNP (2,2′-Dithiobis(5-nitropyridine)), which produced a yellow end product called 2-nitro-5-thiobenzoic acid. A microplate reader was used to measure absorbance at 412 nm. GSH content is expressed in μmole/mg of protein. The level of GST was determined by forming a CDNB conjugate and measuring its absorbance at 340 nm. GST activity is calculated using the extinction coefficient of the product formed and is expressed as μmoles of CDNB conjugate/min/mg of proteins. In the presence of catalase, the degradation of H_2_O_2_ was measured. A microplate reader was used to measure absorbance at 240 nm. Catalase activity is measured in moles H_2_O_2_/min/mg of protein. Malondialdehyde, the end product of LPO, was used to assess its level (MDA). At 532 nm, absorbance was measured using a microplate reader. LPO values are given in TBARS nmoles/min/mg protein [15].

### 4.13. Hematoxylin and Eosin (H&E) Staining and Immunohistochemistry (IHC)

For morphological analysis, five rats were used in each group. Stomach tissues were fixed in 4% paraformaldehyde and embedded in paraffin, till further analysis. Subsequently, the tissues were sectioned at 5 μm by means of a rotary microtome and were stained with hematoxylin and eosin (H&E). According to [33], the stomach tissues were examined using an optical microscope, and photographs were taken. Immunohistochemical staining was carried out. Tissue sections on slides were deparaffinized with three different absolute xylenes and rehydrated with ethyl alcohol in varying concentrations on (from 100 percent [absolute] to 70 percent). After that, the slides were washed with distilled water and maintained for 10 min in 0.01 M phosphate-buffered saline (PBS). Following the antigen retrieval step, the slides were incubated overnight with primary antibody, followed by 2 h of treatment with appropriate biotinylated secondary antibodies, and finally 1 h of treatment with Avidin-biotin complex (ABC) reagents (Standard Vectastain ABC Elite Kit; Vector Laboratories, Burlingame, CA, USA) at optimum room temperature. The sections were washed in PBS and stained with 3,3′-Diaminobenzidine (DAB) solution as a chromogen; they were then washed in distilled water, dehydrated in graded ethanol solutions (70, 95, and 100%), fixed in xylene, then cover-slipped with a mounting media and allowed to air dry. A light microscope (Olympus, Tokyo, Japan) was used to examine the results, which was coupled to a high-quality digital photo-microscopy system. A light microscope was used to obtain immunohistochemical TIF images (5 images per plate). Phosphorylated nuclear factor kappa β (SC-271908 Santa Cruz Biotechnology, Dallas, TX, USA), tumor necrosis factor α (SC-52B83 Santa Cruz Biotechnology, Dallas, TX, USA) and COX-2 (SC-514489 Santa Cruz Biotechnology, Dallas, TX, USA) antibodies were quantified using ImageJ software.

### 4.14. Enzyme-Linked Immunosorbent Assay (ELISA)

Tumor necrotic factor (TNF-α), prostaglandin-E2 (PGE2), interleukin-8 (IL-8), and phosphorylated nuclear factor kappa B (p-NF-κB) detection was conducted according to the manufacturer’s instructions (Elabscience). The stomach tissues (*n* = 3) were kept in at biofreezer (−80 °C) for storage, homogenize at 15 rpm × 1000 using SilentCrusher M (Heidolph) and the supernatant was collected after centrifugation (at 1350× *g* for 1 h). The supernatant was then analyzed for TNF-α (Catalog No: E-EL-R0019), PGE2 (Catalog No: E-EL-0034), IL-8 (Catalog No: EKF57830), and p-NF-ƙb (Catalog No: E-EL-R0674) quantification through Elabscience Rat ELISA kit.

### 4.15. Real Time-Polymerase Chain Reaction (RT-PCR)

After homogenization of gastric tissues (*n* = 3), the trizol method was used to extract total ribonucleic acid (RNA) following the manufacturer’s instructions. Using 1–2 µg of total RNA, cDNA was synthesized by reverse transcriptase enzyme, and cDNA was then amplified by real-time PCR using a thermocycler. The mRNA expression was normalized to expression levels of Beta-actin. The relative gene expression was determined by the 2^−ΔΔCT^ method for real-time quantitative PCR [34]. Primers sequences for β-actin and H^+^/K^+^-ATPase are as follows:

Rat-BetaActin-Forward: CCCGCGAGTACAACCTTCT
Rat-BetaActin-Reverse: CGTCATCCATGGCGAACT
H^+^/K^+^-ATPase Forward: TATGAATTGTACTCAGTGGA
H^+^/K^+^-ATPase Reverse: TGGTCTGGTACTTCTGCT

### 4.16. Toxicity

A total of 10 non-pregnant, nulliparous female rats were used in this study to determine the acute toxicity of plant extract. They were placed into two groups, each with five females: the control group and the treatment group. Only one was given the limited oral dose of 2000 mg/kg in accordance with OECD standards 425 and they were deprived of food and water overnight. The rat was monitored for 24 h and if it survived, the same approach was used on the other rats in the therapy group. They were examined for 48 h for signs of distress, and mortality and then daily for 14 days for various signs of toxicity such as squinting eyes, writhing, salivation, tremors, convulsions, loss of fur, change in overall behavior, stress, and mortality. Blood samples were taken from animals via cardiac puncture on the 15th day for various biochemical analyses such as wet weights of their organs, antioxidant profile, liver function tests, renal function tests, and then animals were eventually sacrificed under anesthesia and vital organs were collected for histopathological examination (31).

### 4.17. In-Silico Studies

The docking studies of rutin were carried out through Auto Dock Vina and PyRX software against H^+^/K^+^-ATPase (PDB ID:4ux2) and calcium channel (PDB ID:1t3s). All the targets were downloaded from the protein data bank (http://www.rcsb.org/pdb/home/home.do; accessed on 1 July 2022) in PDB format purified through the “Discovery Studio Visualizer” (DSV). Three-dimensional structure of standard drugs, i.e., omeprazole (PubChem CID 4594) and verapamil (PubChem CID 2520) were downloaded from PubChem database and then converted to PDBQT format by Auto Dock tools. The results were analyzed as the binding affinities/E-values (kcal/mol) and best binding pose. Post docking analysis via Biovia Discovery Studio Client 2016 was carried out using one best pose with the lowest energy value. 2D images were evaluated to determine the interactions between amino acid residues and ligands with the receptor [30].

### 4.18. Molecular Dynamic (MD) Simulations

All of the top-ranked docked complexes were subjected to molecular dynamics simulations using Amber 18. The simulations were based on docked structures of the protein with an inhibitor. For 50 nanoseconds (ns), simulations were run in a periodic water box with the ff03.r1 force field. The topologies of the study proteins were recorded using the Leap module in Amber 18 tools. To neutralize the systems, sodium (Na^+^) ions were added. The neutralized systems were then solvated with a distance of 8.0 utilizing the water molecules box (TIP3PBOX). The protonation status of the histidine residues in the proteins has been determined. Before running a production run of MD simulations, the solvated systems were completely reduced. For the first 1500 interactions, the steepest descent method utilizing the SANDER module was used, followed by 1000 steps of the conjugate gradient. These 2500 energy minimization cycles were designed to alleviate adverse protein structural conflicts. Every run began with a 100 ps heating session that progressed from 0 K to 300 K while maintaining a pressure of 1 atm. The first round of 100 ps of equilibration at a steady temperature of 300 K is required prior to the production phase. An interchange of kinetic and potential energy occurred during the equilibration phase. Total energy remained nearly constant throughout the equilibration, whereas potential and kinetic energies differed. In order to acquire statistically precise results, equilibration was followed by a production run of 50 ns for both natural and mutant proteins. To limit all atoms covalently linked to a hydrogen atom, the SHAKE algorithm was used. Periodic boundary conditions with canonical ensemble were utilized in the simulation box. The temperature was kept constant using the Berendsen coupling integration procedure with a non-bonded cutoff of 8.0. The Ewald summation method was used to do MD simulations. Chimera and QtGrace were used to conduct post-simulation studies (root-mean square deviation (RMSD) and secondary structure timeline analyses). Molecular Mechanics Poisson Boltzmann surface area and molecular mechanics Generalized Born surface area (MMGB/PBSA) techniques were used to calculate the binding free energy for both protein–inhibitor docked complexes. To compute the binding free energy differences, 1000 snapshots were taken during the MD simulation trajectory using the same methods as before [35].

### 4.19. Statistical Analysis

Data were expressed as Mean ± SEM (*n* = 5) and median effective concentrations (EC50) having 95% confidence intervals. Statistical analysis of the results was analyzed using one-way ANOVA followed by *post-hoc* Tukey’s test. Non-linear regression using the Graph Pad program (GraphPAD, SanDiego, CA, USA) was used to analyze the concentration-response curves. Image J was used to analyze immunohistochemistry images.

## 5. Conclusions

**Figure 20:**
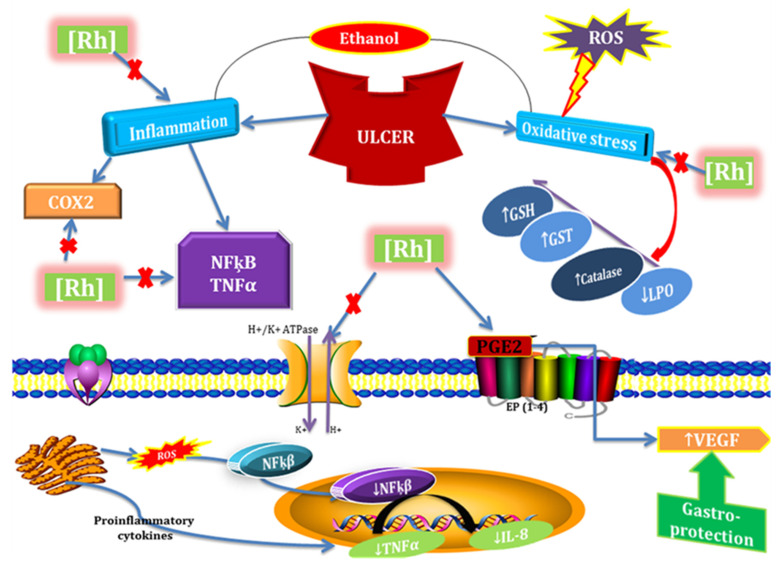
Graphical representation to elaborate the possible mechanism underlying antioxidant, anti-inflammatory, and anti-ulcer potential of *Rumex hastatus* (RH) against ethanol-induced gastric ulcer. ROS (reactive oxygen species), COX2 (cyclooxygenase 2), NFƙB (nuclear factor kappa B), TNF-α (tissue necrosis factor α), IL-8 (interleukin 8), PGE2 (prostaglandins E2), GSH (glutathione), GST (glutathione-S-transferase), LPO (lipid peroxidation), VEGF (vascular endothelial growth factor).

In conclusion, our experimental findings provide substantial evidence that *Rumex hastatus* extracts and its phytoconstituent could be considered potent antioxidant and anti-inflammatory drug candidates that possess anti-diarrheal, anti-secretary, antispasmodic, anti *H. pylori*, and anti-ulcer potential. Furthermore, we demonstrated the involvement of the H^+^/K^+^-ATPase pathway in the gastroprotective activity. It elevates the level of protective GST, GSH, and catalase and down-regulates oxidative stress marker (LPO). It reverses the ethanol induced pathological changes, confirmed by H&E staining and IHC staining of gastric tissues. We also analyzed certain safety aspects of *Rumex hastatus* and it exhibited a relative safety profile as no impairment was observed in kidneys, heart, liver, and brain further assisted by biochemical analysis. Due to multi effective properties, it may reduce polypharmacy, be economically cost-effective, decrease medication error, and drug-drug interactions and be effective in gastrointestinal disorders (Figure 20).

## Figures and Tables

**Table 1:** Phytochemical analysis of *Rumex hastatus* crude extract (Rh.Cr) and fractions: n-hexane (Rh.n-Hex), ethyl acetate (Rh.ETAC) and aqueous (Rh.Aq).

Phytochemical. Constituents	Rh.Cr	Rh.nHex	Rh.ETAC	Rh.Aq
Alkaloids	+	+	+	+
Anthraquinones	+	+	+	−
Cardiac glycosides	+	−	+	−
Coumarins	+	+	−	−
Flavonoids	+	+	+	−
Saponins	+	+	+	+
Tannins	+	−	+	−
Terpenoids	+	−	−	+

+, present; −, absent.

**Table 2:** Gas chromatography-mass spectrometry (GC-MS) analysis of *Rumex hastatus* crude extract (Rh.Cr) and fractions: n-hexane (Rh.n-Hex) and ethyl acetate (Rh.ETAC).

Samples	R.Time	Area	Conc (%)
Rh.Cr			
2-Propylmalonic acid	6.584	14,101	6.42
Alpha-D-Galactopyranose methyl glycoside	16.701	76,145	34.68
Nonanoic acid	16.847	14,703	6.70
Tetradecanoic acid	21.299	10,318	4.70
Tridecanoic acid, methyl ester	24.769	14,889	6.78
n-Hexadecanoic acid	25.406	48,840	22.24
9,12-Octadecadienoic acid, methyl ester	27.993	7974	3.63
11-Octadecenoic acid, methyl ester	28.113	13,588	6.19
3-Tetradecyne	28.660	7751	3.53
10-Undecenal	28.770	11,261	5.13
Octadecanoic acid	29.258	6500	2.88
**Rh.n-Hex**			
Decane	5.994	1,489,269	4.34
Undecane	7.178	379,968	1.11
4-Methylundecane	7.268	406,539	1.18
2-Methylundecane	7.365	746,639	2.17
3-Methyltridecane	7.518	437,725	1.27
Rutin	8.189	7,806,882	18.32
2,6-Dimethylundecene	8.526	1,234,816	3.60
4-Methyltridecane	9.602	627,733	1.83
2-Methylheptadecane	9.716	983,544	2.86
3-Methyldodecane	9.881	621,852	1.81
2-Methyldecane	9.943	760,818	2.22
Heptacosanoic acid, methyl ester	11.158	171,956	0.50
Dodecane	11.843	258,222	0.75
2,4-Dimethylundecane	11.918	216,800	0.63
2-Methyltridecane	12.163	301,156	0.88
3-Methyltridecane	12.334	196,885	0.57
2,6-Dimethylheptadecane	12.491	212,684	0.62
Hexadecane	14.586	236,082	0.69
Tridecanoic acid, methyl ester	16.039	1,944,396	5.66
1-Fluorodecane	17.665	105,610	0.31
Tridecane	17.827	1,019,712	2.97
Methyl tetradecanoate	20.598	1,057,755	3.08
Pentadecanoic acid, 14-methyl-,methyl ester	24.805	8,207,882	23.91
Hexadecanoic acid, ethyl ester	25.784	361,201	1.05
Hexadecanoic acid, 15-methyl-,methyl ester	26.721	164,157	0.48
9,12-Octadecadienoic acid, methyl ester, (E,E)	28.044	5,433,055	15.83
6-Octadecenoic acid, methyl ester, (Z)	28.164	4,485,856	13.07
11-Octadecenoic acid, methyl ester	28.236	208,642	0.61
Phytol	28.367	282,891	0.82
Hexadecanoic, 15-methyl-, methyl ester	28.621	1,546,940	4.51
2-Methyl-Z,Z-3, 13-octadecadienol	29.457	230,898	0.67
**Rh.ETAC**			
Decane	6.004	13,295	3.17
Octanoic acid	7.429	4616	1.10
Dodecane	8.194	9079	2.16
Pentadecanoic acid	16.853	4850	1.16
Phthalic acid, 2-ethylhexyl isohexyl ester	20.368	326,359	77.80
n-Hexadecanoic acid	25.412	38,710	9.23
6-Octadecenoic acid, methyl ester, (Z)	28.108	8104	1.93
Oleic acid	28.777	14,458	3.45

**Table 3:** Effect of *Rumex hastatus* crude extract (Rh.Cr) and fractions: n-hexane (Rh.n-Hex), ethyl acetate (Rh.ETAC), aqueous (Rh.Aq), rutin and loperamide against castor-oil induced diarrhea in mice.

Treatment (mg/kg)	No of Wet Feces	Total No of Feces	Average Weight of Wet Feces (gm)	Average Weight of Total Feces (gm)	% Inhibition of Defecation	% WWFO	% WTFO
Saline (10 mL/kg) + Castor-oil (10 mL/kg)	7.2 ± 0.3	8.4 ± 0.24	0.44 ± 0.05	0.5 ± 0.01	0	0	0
Rh.Cr (50 mg/kg) + Castor-oil (10 mL/kg)	4.2 ± 0.09	6 ± 0.24	0.26 ± 0.06	0.38 ± 0.05	41.6 **	59.09	76
Rh.Cr (100 mg/kg) + Castor-oil (10 mL/kg)	1.4 ± 0.17	4.2 ± 0.1	0.06 ± 0.04	0.25 ± 0.03	80.5 ***	13.6	50
Rh.Cr (300 mg/kg) + Castor-oil (10 mL/kg)	0 ± 0.0	2.5 ± 0.05	0 ± 0.0	0.14 ± 0.04	100 ***	0	28
Rh.n-Hex (50 mg/kg) + Castor-oil (10 mL/kg)	3.9 ± 0.09	6.8 ± 0.2	0.24 ± 0.18	0.39 ± 0.11	45.8 **	54.5	78
Rh.n-Hex (100 mg/kg) + Castor-oil (10 mL/kg)	2.9 ± 0.05	5.5 ± 0.3	0.16 ± 0.27	0.32 ± 0.13	59.7 **	36.36	64
Rh.n-Hex (300 mg/kg) + Castor-oil (10 mL/kg)	1.4 ± 0.18	4 ± 0.06	0.06 ± 0.09	0.23 ± 0.19	80.5 ***	13.6	46
Rh.ETAC (50 mg/kg) + Castor-oil (10 mL/kg)	5.5 ± 0.5	6.2 ± 0.4	0.33 ± 0.1	0.41 ± 0.12	23.6 *	75	82
Rh.ETAC (100 mg/kg) + Castor-oil (10 mL/kg)	4.5 ± 0.24	5.2 ± 0.2	0.29 ± 0.04	0.31 ± 0.03	37.5 **	62	67.2
Rh.ETAC (300 mg/kg) + Castor-oil (10 mL/kg)	2.7 ± 0.23	5.3 ± 0.32	0.13 ± 0.45	0.3 ± 0.04	62.5 ***	29.5	60
Rh.Aq (50 mg/kg) + Castor-oil (10 mL/kg)	7.2 ± 0.08	8.2 ± 0.12	0.44 ± 0.01	0.49 ± 0.05	0	100	98
Rh.Aq (100 mg/kg) + Castor-oil (10 mL/kg)	6.8 ± 0.05	7.5 ± 0.1	0.41 ± 0.06	0.45 ± 0.05	5.55	93.1	90
Rh.Aq (300 mg/kg) + Castor-oil (10 mL/kg)	6.5 ± 0.22	7.2 ± 0.1	0.40 ± 0.03	0.44 ± 0.05	9.72	90.1	88
Rutin (50 mg/kg) + Castor-oil (10 mL/kg)	4.2 ± 0.2	7 ± 0.30	0.27 ± 0.08	0.39 ± 0.05	41.6 **	61.36	78
Rutin (100 mg/kg) + Castor-oil (10 mL/kg)	1.4 ± 0.24	4.5 ± 0.16	0.07 ± 0.04	0.26 ± 0.01	80.5 ***	15.9	52
Rutin (200 mg/kg) + Castor-oil (10 mL/kg)	0 ± 0.0	2.7 ± 0.03	0 ± 0.0	0.15 ± 0.02	100 ***	0	30
Loperamide (2 mg/kg) + Castor-oil (10 mL/kg)	0 ± 0.0	2.2 ± 0.2	0 ± 0.0	0.11 ± 0.03	100 ***	0	22

Values expressed as mean ± SEM (*n* = 5). One-way ANOVA with *post-hoc* Tukey’s test. *p* < 0.001 vs. saline group, * *p* < 0.05, ** *p* < 0.01, *** *p* < 0.001 vs. castor oil group.

**Table 4:** Effect of *Rumex hastatus* crude extract (Rh.Cr) and fractions: n-hexane (Rh.n-Hex), ethyl acetate (Rh.ETAC), aqueous (Rh.Aq), rutin and atropine against castor-oil induced fluid accumulation in mice.

Treatment (mg/kg)	% Inhibition
Saline (10 mL/kg)	89.7
Castor-oil (10 mL/kg)	126.8 ^###^
Rh.Cr (50 mg/kg) + Castor-oil (10 mL/kg)	119 *
Rh.Cr (100 mg/kg) + Castor-oil (10 mL/kg)	105 **
Rh.Cr (300 mg/kg) + Castor-oil (10 mL/kg)	85 ***
Rh.nHex (50 mg/kg) + Castor-oil (10 mL/kg)	120.6
Rh.nHex (100 mg/kg) + Castor-oil (10 mL/kg)	110 **
Rh.nHex (300 mg/kg) + Castor-oil (10 mL/kg)	92.8 ***
Rh.ETAC (50 mg/kg) + Castor-oil (10 mL/kg)	118 *
Rh.ETAC (100 mg/kg) + Castor-oil (10 mL/kg)	99 ***
Rh.ETAC (300 mg/kg) + Castor-oil (10 mL/kg)	78 ***
Rh.Aq (50 mg/kg) + Castor-oil (10 mL/kg)	124
Rh.Aq (100 mg/kg) + Castor-oil (10 mL/kg)	122
Rh.Aq (300 mg/kg) + Castor-oil (10 mL/kg)	118 *
Rutin (50 mg/kg) + Castor-oil (10 mL/kg)	109 **
Rutin (100 mg/kg) + Castor-oil (10 mL/kg)	85 ***
Rutin (200 mg/kg) + Castor-oil (10 mL/kg)	75 ***
Atropine (0.1 mg/kg) + Castor-oil (10 mL/kg)	74.10 ***

Values expressed as mean ± SEM (*n* = 5). One-way ANOVA with *post-hoc* Tukey’s test. ^###^ *p* < 0.001 vs. saline group, *p < 0.05, ** *p* < 0.01, *** *p* < 0.001 vs. castor oil group.

**Table 5:** Effect on *Rumex hastatus* crude extract (Rh.Cr) and fractions: n-hexane (Rh.n-Hex), ethyl acetate (Rh.ETAC), aqueous (Rh.Aq), rutin and atropine on charcoal meal transit time in rats.

Treatment (mg/kg)	Mean Length of Intestine (cm)	Distance Moved by Charcoal (cm)	Peristaltic Index (PI) (%)	% Inhibition
Saline (10 mL/kg)	93	0	0	0
Charcoal (25 mg/kg)	92.6	90	97.1 ^###^	0
Rh.Cr (50 mg/kg) + Charcoal (25 mg/kg)	94	65	69.1	28.83 *
Rh.Cr (100 mg/kg) + Charcoal (25 mg/kg)	92.1	46	49.9	48.6 **
Rh.Cr (300 mg/kg) + Charcoal (25 mg/kg)	94	19	20.21	79.18 ***
Rh.n-Hex (50 mg/kg) + Charcoal (25 mg/kg)	96	78.8	82.08	15.4 *
Rh.n-Hex (100 mg/kg) + Charcoal (25 mg/kg)	94	66.9	71.17	26.70 *
Rh.n-Hex (300 mg/kg) + Charcoal (25 mg/kg)	95	54.4	57.2	41.09 **
Rh.ETAC (50 mg/kg) + Charcoal (25 mg/kg)	84	69	82.14	15.40 *
Rh.ETAC (100 mg/kg) + Charcoal (25 mg/kg)	92.1	41	44.51	54.16 **
Rh.ETAC (300 mg/kg) + Charcoal (25 mg/kg)	85	28	32.94	66.07 ***
Rh.Aq (50 mg/kg) + Charcoal (25 mg/kg)	94	90	95.7	1.44
Rh.Aq (100 mg/kg) + Charcoal (25 mg/kg)	95	86.6	91.1	6.1
Rh.Aq (300 mg/kg) + Charcoal (25 mg/kg)	98	86.6	88	9.37
Rutin (50 mg/kg) + Charcoal (25 mg/kg)	83	71	85.5	11.94 *
Rutin (100 mg/kg) + Charcoal (25 mg/kg)	92	60	65.2	32.85 **
Rutin (200 mg/kg) + Charcoal (25 mg/kg)	94	23.6	25.1	74.1 ***
Atropine (0.1 mg/kg, i.p.) + Charcoal (25 mg/kg)	90.8	16.4	18.06	81.40 ***

Values expressed as mean ± SEM (*n* = 5). One-way ANOVA with post-hoc Tukey’s test. ^###^ *p* < 0.001 vs. saline group, * *p* < 0.05, ** *p* < 0.01, *** *p* < 0.001 vs. charcoal group.

**Table 6:** Effect of *Rumex hastatus* crude extract (Rh.Cr) and fractions: n-hexane (Rh.n-Hex), ethyl acetate (Rh.ETAC), aqueous (Rh.Aq) and rutin against *H. pylori* isolates.

Samples	MIC (mg/mL)	Zone of Inhibition (mm)
Rh.Cr	0	0
Rh.n-Hex	2.5	10.66 ± 0.62
Rh.ETAC	0.6	25.33 ± 0.33
Rh.Aq	0	0
Rutin	0.6	31 ± 0.66

MIC: Minimum inhibitory concentration.

**Table 7:** Effect of *Rumex hastatus* crude extract (Rh.Cr) and fractions: n-hexane (Rh.n-Hex), ethyl acetate (Rh.ETAC), aqueous (Rh.Aq), rutin and omeprazole against an ethanol-induced ulcer in rats.

Treatment (mg/kg)	Ulcer Index	% Inhibition
Saline (10 mL/kg)	0 ± 0.0	-
Ethanol (1 mL/100 g)	10 ± 0.3 ^###^	0
Rh.Cr (50 mg/kg) + Ethanol (1 mL/100 g)	7.0 ± 0.31 ***	30
Rh.Cr (100 mg/kg) + Ethanol (1 mL/100 g)	1.5 ± 0.2 ***	87
Rh.Cr (300 mg/kg) + Ethanol (1 mL/100 g)	0.6 ± 0.24 ***	92
Rh.n-Hex (50 mg/kg) + Ethanol (1 mL/100 g)	5.5 ± 0.05 ***	47
Rh.n-Hex (100 mg/kg) + Ethanol (1 mL/100 g)	2.4 ± 0.20 ***	76
Rh.n-Hex (300 mg/kg) + Ethanol (1 mL/100 g)	0 ± 0.0 ***	100
Rh.ETAC (50 mg/kg) + Ethanol (1 mL/100 g)	6.5 ± 0.25 ***	35
Rh.ETAC (100 mg/kg) + Ethanol (1 mL/100 g)	4.8 ± 0.24 ***	52
Rh.ETAC (300 mg/kg) + Ethanol (1 mL/100 g)	2.8 ± 0.07 ***	72
Rh.Aq (50 mg/kg) + Ethanol (1 mL/100 g)	9.8 ± 0.1	2
Rh.Aq (100 mg/kg) + Ethanol (1 mL/100 g)	10 ± 0.2	1.96
Rh.Aq (300 mg/kg) + Ethanol (1 mL/100 g)	9.6 ± 0.24	5.88
Rutin (50 mg/kg) + Ethanol (1 mL/100 g)	4.52 ± 0.14 ***	56.8
Rutin (100 mg/kg) + Ethanol (1 mL/100 g)	2.86 ± 0.02 ***	73.4
Rutin (200 mg/kg) + Ethanol (1 mL/100 g)	1.26 ± 0.12 ***	89.4
Omeprazole (20 mg/kg) + Ethanol (1 mL/100 g)	0.4 ± 0.04 ***	96.2

Values expressed as mean ± SEM (*n* = 5). One-way ANOVA with post-hoc Tukey’s test ^###^ *p* < 0.001 vs. saline group, *** *p* < 0.001 vs. ethanol group.

**Table 8:** Effect of *Rumex hastatus* crude extract (Rh.Cr) against different organs weight of rats.

Organs	Control	Rh.Cr (2000 mg/kg)
Heart	0.36 ± 0.1 g	0.42 ± 0.4 g
Kidney	1.1 ± 0.1 g	1.14 ± 0.3 g
Liver	6.5 ± 0.3 g	6.9 ± 0.2 g
Brain	1.4 ± 0.2 g	1.49 ± 0.1 g

**Table 9:** E-values (Kcal/mol) (binding energy values) and post-dock analysis of the best conformational pose of rutin and standard drugs with targeted proteins.

	Target Protein	E-Value (Kcal/mol)	No of H Bonds	Binding Residues Forming H Bonds
Rutin	H^+^/K^+^-ATPase	−8.7	11	ARG A:846, ARG A:949, ARG A:844, ARG A:775, ASN A:847, TYR A:1033, TYR A:1032, LYS A:782, PRO A:845
	Voltage-gated L-type calcium channel	−9.4	6	ARG A:276, GLN A:301, PHE A:308, SER A:226, VAL A:224
Omeprazole	H^+^/K^+^-ATPase	−7.8	2	GLN A:110, GLY A:108
Verapamil	Voltage-gated L-type calcium channel	−6.2	2	GLU A:309, SER A:226

## Data Availability

Not applicable.

## Supplementary Materials

The supporting information can be downloaded at: https://www.mdpi.com/article/10.3390/molecules27185919/s1. Table S1. Table presenting molecular mechanics Poisson Boltzmann surface area and molecular mechanics Generalized Born surface area (MMPBSA-MMGBSA) values of rutin-H^+^/K^+^-ATPase; Table S2. Table presenting molecular mechanics Poisson Boltzmann surface area and molecular mechanics Generalized Born surface area (MMPBSA-MMGBSA) values of rutin-voltage gated L-type calcium channel.

## Sample Availability

Not available.
