# Supplementary material for: Pharmacological Basis of Rumex hastatus D. Don in Gastrointestinal Diseases with Focusing Effects on H+/K+-ATPase, Calcium Channels Inhibition and PDE Mediated Signaling: Toxicological Evaluation on Vital Organs
Source: Molecules. 2022 Sep 12;27(18):5919. doi: 10.3390/molecules27185919 (PMC9502566; doi:10.3390/molecules27185919)
Supplement: Supplementary file 1 [file molecules-27-05919-s001.zip › molecules-1879656-supplementary.pdf]

Table S1. Table presenting molecular mechanics Poisson Boltzmann surface area and molecular mechanics Generalized Born surface area (MMPBSA-MMGBSA) values of rutin-H^+/K^+-ATPase.

**MMGBSA**	**Average Values**	**Std. Dev**
VDWAALS	-61.9027	3.1507
EEL	-28.9140	8.5848
EGB	47.3976	7.0177
ESURF	-6.9506	0.2889
∆G gas	-90.8166	8.9731
∆G solv	40.4470	7.0278
∆ TOTAL	-50.3696	4.4032
**MMPBSA**	**Average Values**	**Std. Dev**
VDWAALS	-61.9027	3.1507
EEL	-28.9140	8.5848
EPB	58.4900	6.6887
ENPOLAR	-4.5037	0.1797
∆G gas	-90.8166	8.9731
∆G solv	53.9863	6.6765
**∆TOTAL**	**-36.8304**	**4.9028**

VDWAALS: van der waals EEL: electrostatic interaction EPB/EGB: the electrostatic contribution to the solvation free energy calculated by PB and GB respectively.
ESURF: non-polar contribution to solvation energy using SASA (solvent accessible surface area) for GB.
ENPOLAR: non-polar contribution to solvation energy from repulsive solute-solvent interactions for PB.
Delta G gas: change in energy in gaseous phase.
Delta G solv: change in energy in solvation phase.

Table S2. Table presenting molecular mechanics Poisson Boltzmann surface area and molecular mechanics Generalized Born surface area (MMPBSA-MMGBSA) values of rutin-voltage gated L-type calcium channel.
VDWAALS: van der waals
EEL: electrostatic interaction

**MMGBSA**	**Average values**	**Std.Dev**
VDWAALS	-55.6448	4.0571
EEL	-22.6093	8.1912
EGB	34.7234	7.9352
ESURF	-6.3886	0.2655
ΔGgas	-78.2542	9.1786
ΔG solv	28.3348	7.8232
Δ TOTAL	-49.9194	3.9229
**MMPBSA**	**Average Values**	**Std. Dev**
VDWAALS	-55.6448	4.0571
EEL	-22.6093	8.1912
EPB	45.0120	8.3928
ENPOLAR	-4.3173	0.1342
ΔG gas	-78.2542	9.1786
ΔG solv	40.6947	8.3160
**ΔTOTAL**	**-37.5595**	**3.4529**

EPB/EGB: the electrostatic contribution to the solvation free energy calculated by PB and GB respectively.
ESURF: non-polar contribution to solvation energy using SASA (solvent accessible surface area) for GB.
ENPOLAR: non-polar contribution to solvation energy from repulsive solute-solvent interactions for PB.
Delta G gas: change in energy in gaseous phase.
Delta G solv: change in energy in solvation phase.
